# Urothelium marker UPK2 identifies aggressive colorectal cancers with distinct molecular and histological features

**DOI:** 10.1038/s41416-025-03300-1

**Published:** 2025-12-11

**Authors:** Ville K. Äijälä, Jouni Härkönen, Päivi Sirniö, Tuomo Mantere, Hanna Elomaa, Onni Sirkiä, Akseli Kehusmaa, Henna Karjalainen, Meeri Kastinen, Vilja V. Tapiainen, Maarit Ahtiainen, Olli Helminen, Erkki-Ville Wirta, Jukka Rintala, Sanna Meriläinen, Juha Saarnio, Tero Rautio, Toni T. Seppälä, Jan Böhm, Jukka-Pekka Mecklin, Anne Tuomisto, Markus J. Mäkinen, Juha P. Väyrynen

**Affiliations:** 1 Translational Medicine Research Unit, University of Oulu, and Medical Research Center Oulu, Oulu University Hospital, Oulu, Finland; 2 Department of Pathology, Hospital Nova of Central Finland, Well Being Services County of Central Finland, Jyväskylä, Finland; 3 Faculty of Health Sciences, A.I. Virtanen Institute for Molecular Sciences, University of Eastern Finland, Kuopio, Finland; 4 Laboratory of Cancer Genetics and Tumor Biology, Translational Medicine Research Unit, Medical Research Center Oulu and Biocenter Oulu, University of Oulu, Oulu, Finland; 5 Research Program in Systems Oncology, University of Helsinki, Helsinki, Finland; 6 Department of Environmental and Biological Sciences, University of Eastern Finland, Kuopio, Finland; 7 Central Finland Biobank, Hospital Nova of Central Finland, Well Being Services County of Central Finland, Jyväskylä, Finland; 8 Department of Surgery, Oulu University Hospital, Oulu, Finland; 9 Department of Gastroenterology and Alimentary Tract Surgery, Tampere University Hospital, Tampere, Finland; 10 Faculty of Medicine and Health Technology, Tampere University and Tays Cancer Centre, Tampere University Hospital, Tampere, Finland; 11 Department of Gastrointestinal Surgery, Helsinki University Central Hospital, University of Helsinki, Helsinki, Finland; 12 Applied Tumor Genomics, Research Program Unit, University of Helsinki, Helsinki, Finland; 13 Department of Education and Research, Well Being Services County of Central Finland, Jyväskylä, Finland; 14 Faculty of Sport and Health Sciences, University of Jyväskylä, Jyväskylä, Finland

**Keywords:** Translational research, Prognostic markers, Colorectal cancer

## Abstract

**Background:**

Uroplakin-2 (UPK2) is a relatively specific marker for urothelial cancer, often used in the differential diagnosis of tumors of unknown origin. UPK2 expression has been observed in colorectal cancers (CRCs), prompting further investigation.

**Methods:**

UPK2 expression was analyzed in two independent CRC cohorts (*N* = 1851) and The Cancer Genome Atlas (*N* = 467). We investigated the histopathological, immunological, molecular, and clinical characteristics of UPK2-positive CRCs.

**Results:**

UPK2 was expressed in 12% of CRCs and associated with adverse features including advanced stage, lymphovascular invasion, tumor budding, and micropapillary growth (*p* < 0.01). UPK2 positivity correlated with higher CRC-specific mortality in both cohorts (Cohort 1: HR 1.97, 95% CI 1.00–3.88; Cohort 2: HR 3.33, 95% CI 2.15–5.16). In the larger cohort, this association remained independent of other prognostic parameters (HR 2.31, 95% CI 1.46–3.65). UPK2-positive tumors showed reduced infiltration of CD3 + T cells, B cells, plasma cells, and M2-like macrophages. Molecularly, these tumors were associated with *TP53* mutation, CMS4 subtype, and upregulation of genes linked to keratinization and squamous differentiation, such as *KRT17* and *DSG3* (*p* < 0.01).

**Conclusions:**

UPK2 marks a distinct subset of CRCs with poor prognosis, epithelial-mesenchymal transition, micropapillary growth, and squamous differentiation. These findings may affect the development of targeted therapies in precision medicine.

## Introduction

Colorectal cancer (CRC) ranks as the second leading cause of cancer-related deaths worldwide [1]. The treatment protocols and prognosis of CRC are influenced by various factors, such as the Tumor-Node-Metastasis classification, mismatch-repair (MMR) status, and tumor morphology [2–4]. Around 20% of patients present with metastatic disease at diagnosis, and in cases where the tumor is unresectable, systemic therapies like chemoradiotherapy, immunotherapy, and biologic therapies serve as the primary treatment approaches [5]. Consequently, molecular profiling of CRC tumors has become crucial for refining classification systems and guiding precision oncology. For instance, targeted therapies such as Bevacizumab and Cetuximab, monoclonal antibodies against VEGF-A and EGFR, are routinely used for the treatment of metastatic CRC [6].

In a recent study, we characterized the features of micropapillary CRC, a relatively rare and aggressive morphological subtype, and noted *UPK2* expression in a subset of these tumors [7]. UPK2 is a component of urothelial plaques that influence the permeability barrier in urothelial umbrella cells [8]. Clinically, it serves as a relatively specific and sensitive immunohistochemical marker for urothelial carcinomas, helping to distinguish them from adenocarcinomas of other origins [9]. However, the functional and prognostic implications of UPK2 expression in urothelial and other cancers remains poorly understood. To our knowledge, aside from our recent report focusing on micropapillary CRC [7], no prior studies have reported UPK2 protein expression in CRCs, and the prognostic significance of UPK2 expression in CRC and the molecular characteristics of UPK2-positive CRCs remain unexplored.

The purpose of this study was to evaluate the prevalence and prognostic significance of UPK2 expression in CRC, as well as to characterize its histopathological, immunological, and molecular correlates.

## Methods

### Study population

Three independent cohorts were analyzed in this study: two from Finnish hospitals and one from The Cancer Genome Atlas (TCGA) dataset.

Cohort 1 included 1011 CRC patients who underwent surgery at Oulu University Hospital between 2006 and 2020. Cohort 2 comprised 1343 CRC patients who were surgically treated at Central Finland Central Hospital between 2000 and 2015. For molecular characterization of CRCs with *UPK2* expression, data from the TCGA cohort (*N* = 467) were utilized.

Patients who had undergone preoperative radiotherapy or chemoradiotherapy were excluded from the analyses (Cohort 1: *N* = 235; Cohort 2: *N* = 243) due to the potential impact of neoadjuvant treatments on tumor histology [10]. Additionally, patients who died within 30 days of surgery were excluded from survival analyses (Cohort 1: *N* = 5; Cohort 2: *N* = 37). Consequently, 776 patients in Cohort 1 and 1,100 patients in Cohort 2 were included in overall analyses, while 771 patients in Cohort 1 and 1063 patients in Cohort 2 were included in survival analyses. Comparative analyses revealed several statistically significant differences between Cohorts 1 and 2, including younger patient age, later operation years, more frequent rectal location, lower grade, lower disease stage, more frequent lymphovascular invasion, and more frequent tumor budding in Cohort 1 (Table S1).

### Histopathologic analysis

Histological parameters were analyzed using whole-slide digital images of hematoxylin & eosin (H&E)-stained tumor sections. Disease stage was determined according to the UICC (Union for International Cancer Control) criteria [11]. Tumor grading was based on WHO criteria, classifying tumors as either low-grade or high-grade [12]. The micropapillary growth pattern was evaluated as previously described [7]. Tumor budding was assessed at the invasive margin following guidelines from the International Tumor Budding Consensus Conference [13]. Lymphovascular invasion was defined as the presence of tumor cells within vascular spaces. Lymphocytic reaction patterns, including tumor-infiltrating lymphocytes, intratumoral periglandular reaction, peritumoral reaction, and Crohn’s-like lymphoid reaction, were assessed according to the criteria established by Ogino et al. [14].

### Immunohistochemistry and in situ hybridization

Tissue microarrays were employed for immunohistochemistry, designed to include four 1 mm-diameter cores from each tumor, two from the tumor center and two from the invasive margin [15, 16]. UPK2 immunohistochemistry was performed with Leica BOND RX stainer using BC21 antibody (Biocare ACI3051C; 1:50, 30 min), coupled with heat-induced epitope retrieval using the BOND Epitope Retrieval Solution 2 (Leica AR9640, 30 min, 100 °C) and detection using the BOND Polymer Refine Detection kit (Leica DS9800). RNA in situ hybridization was performed with Leica BOND RX using the RNAscope™ 2.5 LS Reagent Kit-BROWN (Advanced Cell Diagnostics 322100) and the following probes: *UPK2* (Advanced Cell Diagnostics 405818) and *PPIB* (Advanced Cell Diagnostics 313908).

**Fig. 1:**
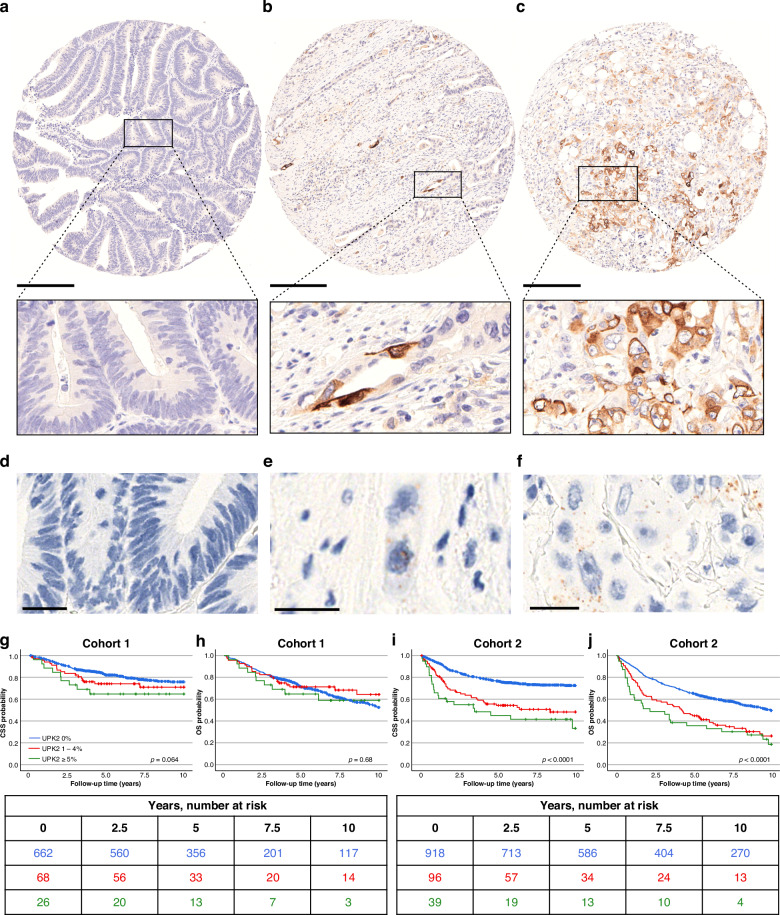
Immunohistochemistry, in situ hybridization and Kaplan-Meier survival analyses of uroplakin-2. **a**–**c** Example immunohistochemistry images and close-ups of UPK2-negative (**a**), UPK2-low (**b**), and UPK2-high colorectal cancer. UPK2 expression is primarily cytoplasmic and limited to tumor cells. Examples of *UPK2* mRNA in situ hybridization in UPK2-negative (**d**), UPK2-low (**e**), and UPK2-high (**f**) colorectal cancers. *UPK2* mRNA is indicated by brown dots. The association of UPK2 expression with cancer-specific survival (**g**) and overall survival (**h**) in Cohort 1. The association of UPK2 expression with cancer-specific survival (**i**) and overall survival (**j**) in Cohort 2. Scalebars for (**a**–**c**), 250 µm; for D-F, 25 µm. CSS cancer-specific survival, OS overall survival.

UPK2 expression was primarily cytoplasmic (Fig. 1a–c) and was visually assessed based on the percentage of positive tumor cells. Evaluations were performed blinded to the study endpoints. Given the low and focal nature of UPK2 staining in CRC and the absence of a validated clinical cutoff, tumors were categorized into groups of 0%, 1–4%, and ≥5% for downstream analyses. The ≥5% threshold for high UPK2 expression was chosen to represent a clearly discernible focus of staining on microscopy and to reduce the influence of rare single-cell positivity. Spatial heterogeneity was assessed in Cohort 1 by scoring UPK2 immunohistochemistry separately in paired tissue microarray cores from the tumor center and the invasive front. The reproducibility of UPK2 evaluation was assessed in 57 cases by VKÄ and JPV. Similar evaluation was also performed for L1CAM expression and KRT17 expression. In situ hybridization for *UPK2* mRNA was performed for a tissue microarray of 57 tumors (Fig. 1d–f). Tumors were evaluated based on the percentage of tumor cells with any hybridization signals, as well as the average number of hybridization signals per cell. To ensure RNA quality, tumors with *PPIB* (positive control probe) signal count per cell less than 5 (*N* = 5) were excluded from analysis.

Additional proteins examined using immunohistochemistry included L1CAM (BioLegend, clone 14.10, 1:50), DSG3 (Abcam, clone EPR14101, 1:80), KRT17 (Leica, clone E3, 1:20), TP53 (Epredia, clone DO-7, 1:200), and MUC16 (CA125) (BioLegend, clone 618 F, 1:100). These stainings were performed with Leica BOND RX or Leica BOND-III automated stainer using the BOND Polymer Refine Detection kit (Leica DS9800) and BOND Epitope Retrieval Solution 2 (Leica AR9640, 30 min, 100 °C). For L1CAM (membranous), MUC16 (membranous), DSG3 (membranous), and KRT17 (cytoplasmic) expression, the percentage of positive tumor cells was visually assessed. Tumors with positive tumor cell percentage >0% were considered positive for these markers. *TP53* status, MMR status, and *BRAF* V600E mutation status of the tumors were evaluated as described earlier [7, 15, 17].

### Immune cells

Immune cell densities in tumor intraepithelial and stromal compartments in tissue microarrays of Cohort 2 were evaluated using multiplex immunohistochemistry and digital image analysis. The staining process employed a cyclic method with 3-Amino-9-ethylcarbazole as the chromogen [18]. For this study, the following cell types were included: CD3^+^ T cells, CD20^+^CD79A^+^ B cells, CD20^-^CD79A^+^ plasma cells, M1-like and M2-like macrophages, CD14^+^HLA-DR^+^ mature monocytic cells, CD14^+^HLA-DR^-^ immature monocytic cells, CD66B^+^ granulocytes, and tryptase^+^ mast cells. Macrophage classification was based on a polarization index incorporating four polarization markers—CD86 and HLA-DR for M1 and CD163 and CD206 for M2 [19]. Antibody performance and working dilutions were first established by conventional single-plex immunohistochemistry on a tissue microarray containing tonsil, normal colonic mucosa, and colorectal adenocarcinoma cores. The cyclic multiplex immunohistochemistry protocols were then optimized and verified by demonstrating concordant staining patterns between multiplex and single-plex immunohistochemistry. During image review, marker positivity was accepted only when colocalized with hematoxylin-positive nuclei and appropriate immune cell morphology. Additional details on panel validation are described in Elomaa et al. [18, 19], and Sirkiä et al. [20].

### Bioinformatics

Clinical, gene expression, methylation, copy number variation, and mutation data from The Cancer Genome Atlas (TCGA) were obtained from the NIH GDC PanCanAtlas open-access portal (https://gdc.cancer.gov/about-data/publications/pancanatlas), and RSEM count data for consensus molecular subtyping (CMS) was downloaded from the Broad Institute’s data repository (https://gdac.broadinstitute.org/runs/stddata__2016_01_28/). Micropapillary histology for TCGA specimens was categorized as described earlier [7] (Supplementary File 1). The TCGA cohort included 629 CRC patients. Copy number gain of the *UPK2* locus was evaluated from cases with both SNP array and RNA-seq (EB + +) -data from primary tumor samples (*N* = 485), while other analyses included samples that had both RNA-seq and mutation data (at least one entry in the maf-file) (*N* = 467). The distribution of *UPK2* mRNA levels in the TCGA cohort is presented in Fig. S1. The cutoff for tumors considered UPK2-positive was set at the first local minimum separating the main distribution from the right-hand tail, to demarcate clearly elevated expression from background.

Tumor mutational burden (TMB) was calculated as the number of somatic mutations per 1,000,000 bases, assuming a 30 Mb exome size. CMS classification was performed using CMScaller [21]. MLH1-methylation was defined as the maximum beta-value from *MLH1*-associated probes. CIBERSORT (v0.1.0) was used to deconvolute immune cells from linear bulk mRNA expression using default parameters without quantile normalization.

Differential expression analysis was conducted with limma (v.3.58.1) as described in the user manual (Linear Models for Microarray and RNA-Seq Data User’s Guide page 71, 22/04/23). Protein-protein association analysis was performed with the STRING database [22], using a minimum interaction score of 0.4 (medium confidence). Input comprised the fifty most differentially expressed genes between *UPK2*-positive and *UPK2*-negative tumors in TCGA COAD/READ. Secondary analysis involved UPK2 as the only input to examine associations on a broad scale. Tertiary analysis included the first 50 epithelial-mesenchymal transition involved genes from the EMTome database [23]. Networks were visualized with edge color indicating type of evidence. Because STRING aggregates heterogeneous evidence sources, results are interpreted as associations rather than experimentally validated interactions. Gene set enrichment analysis for GO biological processes was performed using fGSEA (v.1.28.0) [24, 25]. All bioinformatic analyses were performed with R version 4.3.3, and visualization was conducted with ggplot2 (v.3.5.0) and ComplexHeatmap (v.2.18.0).

Copy number variation was analyzed using GISTIC version 2.0.23. Segmented copy number variation data from the PanCanAtlas portal was used as input. GISTIC2 was run with the following parameters: genegistic 1, -rx 0, conf 0.75, -armpeel 1 and gcm extreme using hg19 reference genome. Overrepresentation of copy number gain in the UPK2-locus was assessed with Fisher’s exact test.

### Optical genome mapping

Optical genome mapping experiments were conducted for 35 tumors in accordance with the manufacturer’s instructions using the Saphyr instrument and DLE-1 chemistry (Bionano Genomics, San Diego, CA, USA), as previously described [7]. The data analysis was conducted using the rare variant pipeline included in Bionano Solve software (Bionano Genomics, v.3.8) and visualized by using the Bionano Access software (Bionano Genomics, v.1.8.1). Default confidence scores and size cutoffs were applied for structural variant, aneuploidy, and copy number variant calling. Analysis was focused on rare structural variants (absent from the control database provided by Bionano Genomics) and copy number variants overlapping or in the vicinity of the *UPK2* locus (11q23.3). Additional analyses were performed for regions frequently showing copy number variation in CRC (8q, 13q, and 20q gains; 8p, 15q, 17p, and 18q losses), with cutoff for positivity defined as 5Mbp or larger copy number variation in the locus.

### Statistical analysis

Statistical analyses were conducted using IBM SPSS Statistics for Windows (IBM Corp. version 29.0) or R statistical programming (v.4.3.3). A two-sided *p* < 0.05 was considered statistically significant.

To examine the relationships between UPK2 expression categories and various tumor and patient characteristics, crosstabulation was employed, with statistical significance assessed using the Chi-square test. Wilcoxon signed ranks test was used to evaluate the difference of UPK2 expression in the tumor center and invasion front. Survival outcomes, including cancer-specific survival (CSS) and overall survival (OS) were analyzed using the Kaplan-Meier method and Cox proportional hazards regression models. CSS was the primary measured outcome and OS secondary. CSS was defined as the time from surgery to CRC-related death or the end of follow-up, while OS was defined as the time from surgery to death from any cause or the end of follow-up. The follow-up time was capped at 10 years, given that the majority of CRC deaths occur within the first decade post-surgery. The median follow-up time for censored cases was 7.0 years (IQR 4.7–10.0) in Cohort 1 and 10.0 years (IQR 7.3–10.0) in Cohort 2. There were 277 deaths (153 cancer deaths) in Cohort 1 and 527 deaths (293 cancer deaths) in Cohort 2. Multivariable Cox proportional hazards regression models were adjusted for the following covariates: age ( < 65, 65–75, >75), sex (male, female), year of operation (Cohort 1: 2006–2010, 2011–2015, 2016–2020; Cohort 2: 2000–2005, 2006–2010, 2011–2015), tumor location (proximal colon, distal colon, rectum), disease stage (I–II, III, IV), lymphovascular invasion (no, yes), MMR status (MMR proficient, MMR deficient), and *BRAF* status (wild-type, mutant). Cases with missing data for *BRAF* status (2 cases in Cohort 2) were grouped with the wild-type category to limit degrees of freedom.

In TCGA bioinformatics analyses, Fisher’s exact test was used to compare categorical variables, while the Mann–Whitney U test was applied to differences between categorical and continuous variables. For gene expression analysis, statistical significance was determined using false discovery rate (FDR) values (Benjamini-Hochberg correction).

## Results

### UPK2 is expressed by around 12% of colorectal cancers with aggressive histomorphological features

UPK2 expression was assessed via immunohistochemistry in 1851 CRC cases across two independent cohorts (Table 1). Focal cytoplasmic UPK2 expression was observed in a subset of tumors [94 cases (12.4%) in Cohort 1; 140 cases (12.8%) in Cohort 2] (Fig. 1a–c). In UPK2-positive cases, the median UPK2-positive tumor cell percentage was 2% (IQR: 1–5%) in Cohort 1 and 2% (IQR: 1–5%) in Cohort 2 and ranged from 1 to 60% in both cohorts. To study spatial distribution, UPK2 expression was compared between the tumor center and invasive margin in Cohort 1. No significant difference was observed (*p* = 0.15), indicating comparable staining across compartments. UPK2-positive tumor cell percentage was classified as negative (0%), low (1–4%), and high ( ≥ 5%) for subsequent analyses.

**Table 1:** Baseline patient and tumor characteristics and their associations with UPK2 expression in Cohorts 1 and 2.

	Cohort 1					Cohort 2				
		UPK2					UPK2			
Characteristic	Total N	0%	1–4%	≥5%	*p* value	Total N	0%	1–4%	≥5%	*p* value
All cases	761 (100%)	667 (88%)	68 (8.9%)	26 (3.4%)		1090 (100%)	950 (87%)	101 (9.2%)	39 (3.6%)	
Sex					0.92					0.10
Female	358 (47%)	312 (87%)	33 (9.2%)	13 (3.6%)		539 (49%)	481 (89%)	40 (7.4%)	18 (3.3%)	
Male	403 (53%)	355 (88%)	35 (8.7%)	13 (3.2%)		551 (51%)	469 (85%)	61 (11%)	21 (3.8%)	
Age (years)					0.30					0.38
<65	229 (30%)	204 (89%)	18 (7.9%)	7 (3.1%)		285 (26%)	240 (84%)	33 (12%)	12 (4.2%)	
65–75	280 (37%)	238 (85%)	33 (12%)	9 (3.2%)		381 (35%)	331 (87%)	36 (9.4%)	14 (3.7%)	
>75	252 (33%)	225 (89%)	17 (6.7%)	10 (4.0%)		424 (39%)	379 (89%)	32 (7.5%)	13 (3.1%)	
Year of operation					0.81					0.26
2000–2005	–	–	–	–		339 (31%)	298 (88%)	28 (8.3%)	13 (3.8%)	
2006–2010	151 (20%)	133 (88%)	15 (9.9%)	3 (2.0%)		351 (32%)	314 (89%)	29 (8.3%)	8 (2.3%)	
2011–2015	212 (28%)	185 (87%)	18 (8.5%)	9 (4.2%)		400 (37%)	338 (85%)	44 (11%)	18 (4.5%)	
2016–2020	398 (52%)	349 (88%)	35 (8.8%)	14 (3.5%)		–	–	–	–	
Tumor location					0.76					0.60
Proximal colon	319 (42%)	281 (88%)	26 (8.2%)	12 (3.8%)		530 (49%)	460 (87%)	50 (9.4%)	20 (3.8%)	
Distal colon	204 (27%)	182 (89%)	17 (8.3%)	5 (2.5%)		402 (37%)	355 (88%)	32 (8.0%)	15 (3.7%)	
Rectum	238 (31%)	204 (86%)	25 (11%)	9 (3.8%)		158 (14%)	135 (85%)	19 (12%)	4 (2.5%)	
WHO grade					0.014					0.0056
Low	651 (86%)	579 (89%)	54 (8.3%)	18 (2.8%)		876 (80%)	776 (89%)	69 (7.9%)	31 (3.5%)	
High	110 (14%)	88 (80%)	14 (13%)	8 (7.3%)		214 (20%)	174 (81%)	32 (15%)	8 (3.7%)	
UICC disease stage					0.0010					<0.0001
I	175 (23%)	164 (94%)	8 (4.6%)	3 (1.7%)		182 (17%)	165 (91%)	13 (7.1%)	4 (2.2%)	
II	252 (33%)	230 (91%)	17 (6.7%)	5 (2.0%)		406 (37%)	371 (91%)	27 (6.7%)	8 (2.0%)	
III	250 (33%)	208 (83%)	29 (12%)	13 (5.2%)		352 (32%)	302 (86%)	35 (9.9%)	15 (4.3%)	
IV	84 (11%)	65 (77%)	14 (17%)	5 (6.0%)		150 (14%)	112 (75%)	26 (17%)	12 (8.0%)	
T					0.092					0.080
T1-T2	216 (28%)	198 (92%)	12 (5.6%)	6 (2.8%)		223 (20%)	204 (91%)	15 (6.7%)	4 (1.8%)	
T3-T4	545 (72%)	469 (86%)	56 (10%)	20 (3.7%)		867 (80%)	746 (86%)	86 (9.9%)	35 (4.0%)	
N					<0.0001					<0.0001
N0	441 (58%)	406 (92%)	27 (6.1%)	8 (1.8%)		621 (57%)	566 (91%)	41 (6.6%)	14 (2.3%)	
N1-N2	320 (42%)	261 (82%)	41 (13%)	18 (5.6%)		469 (43%)	384 (82%)	60 (13%)	25 (5.3%)	
M					0.011					<0.0001
M0	677 (89%)	602 (89%)	54 (8.0%)	21 (3.1%)		940 (86%)	838 (89%)	75 (8.0%)	27 (2.9%)	
M1	84 (11%)	65 (77%)	14 (17%)	5 (6.0%)		150 (14%)	112 (75%)	26 (17%)	12 (8.0%)	
Lymphovascular invasion					<0.0001					0.0057
No	416 (55%)	386 (93%)	25 (6.0%)	5 (1.2%)		850 (78%)	753 (89%)	66 (7.8%)	31 (3.6%)	
Yes	345 (45%)	281 (81%)	43 (12%)	21 (6.1%)		240 (22%)	197 (82%)	35 (15%)	8 (3.3%)	
Micropapillary growth pattern					<0.0001					<0.0001
0%	693 (91%)	625 (90%)	53 (7.6%)	15 (2.2%)		993 (91%)	887 (89%)	74 (7.5%)	32 (3.2%)	
1–4%	30 (3.9%)	21 (70%)	4 (13%)	5 (17%)		27 (2.5%)	21 (78%)	4 (15%)	2 (7.4%)	
≥5%	38 (5.0%)	21 (55%)	11 (29%)	6 (16%)		70 (6.4%)	42 (60%)	23 (33%)	5 (7.1%)	
Tumor budding					<0.0001					<0.0001
Grade 1 (0–4)	528 (69%)	490 (93%)	28 (5.3%)	10 (1.9%)		818 (75%)	742 (91%)	54 (6.6%)	22 (2.7%)	
Grade 2 (5–9)	128 (17%)	103 (80%)	18 (14%)	7 (5.5%)		155 (14%)	121 (78%)	26 (17%)	8 (5.2%)	
Grade 3 ( ≥ 10)	105 (14%)	74 (70%)	22 (21%)	9 (8.6%)		117 (11%)	87 (74%)	21 (18%)	9 (7.7%)	
MMR status					0.0003					<0.0001
Proficient	639 (84%)	548 (86%)	65 (10%)	26 (4.1%)		926 (85%)	790 (85%)	98 (11%)	38 (4.1%)	
Deficient	122 (16%)	119 (98%)	3 (2.5%)	0 (0.0%)		164 (15%)	160 (98%)	3 (1.8%)	1 (0.6%)	
*BRAF* status^a^					0.31					0.35
Wild-type	654 (86%)	568 (87%)	62 (9.5%)	24 (3.7%)		908 (83%)	788 (87%)	89 (9.8%)	31 (3.4%)	
Mutant	107 (14%)	99 (93%)	6 (5.6%)	2 (1.9%)		180 (17%)	160 (89%)	12 (6.7%)	8 (4.4%)	

*UICC* Union for International Cancer Control, *MMR* mismatch repair.

^a^BRAF data missing for 2 patients in Cohort 2.

To further confirm UPK2 expression in CRC, in situ hybridization for *UPK2* mRNA was successfully performed for 52 tumors (Fig. 1d–f). A strong correlation (Spearman R = 0.71) was observed between the percentage of UPK2-positive tumor cells detected using immunohistochemistry and mRNA in situ hybridization, providing orthogonal validation for the UPK2 antibody (clone BC21) and demonstrating that UPK2 is expressed in a subset of CRCs at both mRNA and protein level. To assess the reproducibility of UPK2 immunohistochemistry scoring, we calculated κ scores across 57 cases between two investigators. UPK2 evaluation demonstrated substantial agreement (κ = 0.75), comparable to L1CAM (κ = 0.67) and KRT17 (κ = 0.86).

In both cohorts, UPK2 expression was associated with high tumor grade, advanced disease stage, nodal and distant metastases, lymphovascular invasion, micropapillary growth pattern, tumor budding, and MMR proficiency (*p* < 0.05 for all) (Table 1).

### UPK2 expression is associated with poor survival

Considering the association of UPK2 expression with adverse prognostic features, we assessed its impact on survival outcomes. In univariable analyses, UPK2 expression was associated with shorter CSS in both cohorts. The HR for high (vs. negative) UPK2 expression was 1.97 (95% CI 1.00–3.88) in Cohort 1 and 3.33 (95% CI 2.15–5.16) in Cohort 2 (Fig. 1G–J, Table 2).

**Table 2:** Cox proportional hazards regression models for colorectal cancer-specific survival and overall survival according to UPK2 expression in Cohorts 1 and 2.

		Cancer-specific survival			Overall survival		
Variable	No. of cases	No. of events	Univariable HR (95% CI)	Multivariable HR (95% CI)	No. of events	Univariable HR (95% CI)	Multivariable HR (95% CI)
**Cohort 1**							
*UPK2*							
Negative (0%)	662	126	1 (referent)	1 (referent)	246	1 (referent)	1 (referent)
Low (1–4%)	68	18	1.42 (0.87–2.32)	0.63 (0.37–1.06)	21	0.84 (0.54–1.32)	0.55 (0.35–0.89)
High ( ≥ 5%)	26	9	1.97 (1.00–3.88)	1.28 (0.64–2.56)	10	1.14 (0.61–2.15)	0.85 (0.45–1.63)
p_trend_			0.021	0.60		0.89	0.069
**Cohort 2**							
*UPK2*							
Negative (0%)	918	227	1 (referent)	1 (referent)	431	1 (referent)	1 (referent)
Low (1–4%)	96	44	2.29 (1.66–3.16)	1.63 (1.15–2.31)	66	1.92 (1.45–2.49)	1.54 (1.17–2.03)
High ( ≥ 5%)	39	22	3.33 (2.15–5.16)	2.31 (1.46–3.65)	30	2.47 (1.71–3.58)	1.83 (1.25–2.68)
p_trend_			<0.0001	<0.0001		<0.0001	<0.0001

p_trend_ values were calculated by using the three UPK2 categories as continuous variables in univariable and multivariable Cox proportional hazard regression models.

In multivariable analysis, UPK2 expression remained independently associated with shorter CSS in Cohort 2 (p_trend_<0.0001), but not in Cohort 1 (p_trend_=0.60). The multivariable HR for high (vs. negative) UPK2 expression was 1.28 (95% CI 0.64–2.56) in Cohort 1 and 2.31 (95% CI 1.46–3.65) in Cohort 2 (Table 2, Table S2). UPK2 expression was also independently associated with shorter OS in Cohort 2 (p_trend_<0.0001) but not in Cohort 1 (p_trend_=0.069). To directly compare the prognostic utility of UPK2 against a clinically validated biomarker, we evaluated tumor budding (Table S3). UPK2 positivity showed prognostic performance comparable to tumor budding in Cohort 2, but not in Cohort 1.

### UPK2 expression is associated with weaker lymphocytic infiltration

**Fig. 2:**
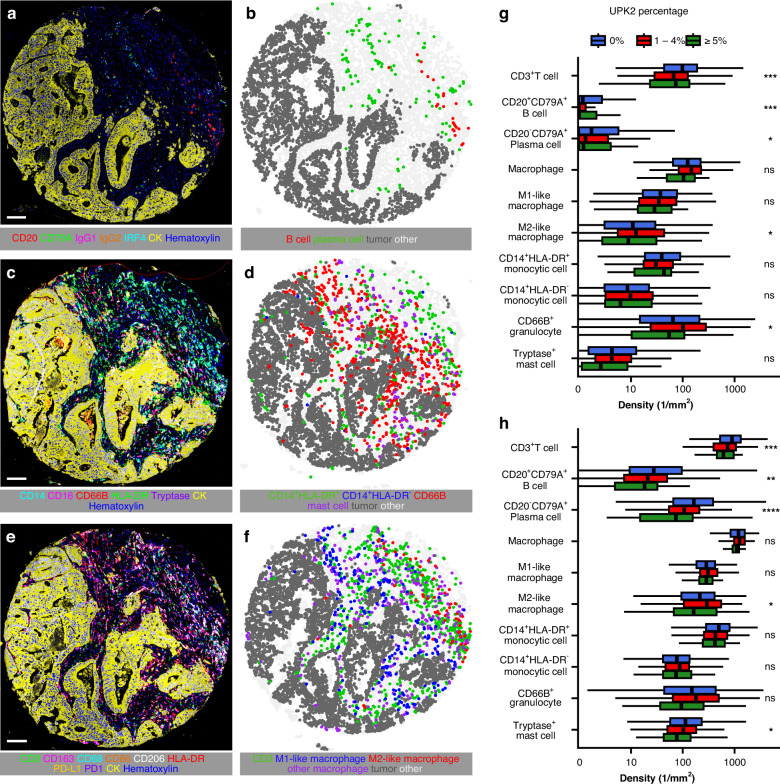
Multiplex-immunohistochemistry, image analysis, and immune cell density analysis for UPK2-positive colorectal cancer. Example multiplex immunohistochemistry images of a tumor (**a**, **c**, and **e**) along with their corresponding cell maps (**b**, **d**, and **f**), generated through machine learning-assisted image analysis. Boxplots showing the distribution of immune cell densities in the tumor intraepithelial region (**g**) and stroma (**h**) based on UPK2 expression. The analyses are based on Cohort 2: *N*  =  1059 for CD3^+^ T-cells; *N*  =  1064 for CD20^+^CD79A^+^ B cells and CD20^−^CD79A^+^ plasma cells; *N* = 1059 for macrophages, M1-like macrophages, and M2-like macrophages; *N*  =  1039 for CD14^+^HLA-DR^+^ mature monocytic cells, CD14^+^HLA-DR^−^ immature monocytic cells, CD66B^+^ granulocytes, and tryptase^+^ mast cells. *p value < 0.05, **p value < 0.01, ***p value < 0.001, ****p value < 0.0001. Scale bars, 100 μm.

To explore the tumor microenvironment composition in UPK2-positive CRC, we focused on MMR proficient tumors, as UPK2-positive CRCs were primarily MMR proficient [3 (2.5%) and 4 (2.4%) MMR deficient cases in Cohorts 1 and 2, respectively]. UPK2-positive CRCs were associated with weaker lymphocytic reactions, as evaluated from hematoxylin & eosin sections, although the association with Crohn’s-like lymphoid reaction did not reach statistical significance (*p* = 0.051) (Table S4). In more detailed analysis using three multiplex immunohistochemistry assays, UPK2-positive CRCs were associated with decreased CD3^+^ T cell, CD20^+^CD79A^+^ B cell, CD20-CD79A^+^ plasma cell, and M2-like macrophage densities in both tumor epithelial and stromal compartments (Fig. 2 and Fig. S2). Associations with T cell, B cell, and plasma cell densities were validated using conventional immunohistochemistry in Cohort 1 with T cells and stromal plasma cells reaching statistical significance (Table S5). Reduced lymphocytic infiltration was also validated through CIBERSORT deconvolution analysis of TCGA cases, showing decreased levels of CD4+ and CD8 + T cells in UPK2-positive cases (Fig. S3). Taken together, these data suggest a weaker antitumor immune response in UPK2-positive tumors.

### UPK2-positive colorectal cancers frequently show *TP53* mutation and gene expression profiles linked to epithelial-to-mesenchymal transition and differentiation towards squamous epithelia

To explore potential mechanisms underlying aberrant UPK2 expression in CRC, we conducted optical genome mapping of 35 tumor samples, including 12 UPK2-positive tumors. We specifically examined the chromosomal locus 11q23.3, which harbors the *UPK2* gene, for structural aberrations. Among the UPK2-positive tumors, 4 cases (33%) showed a copy number gain encompassing the *UPK2* locus (Fig. S4). In contrast, gain of this region was observed only in 1 of 23 (4.3%) UPK2-negative cases (*p* = 0.020). Three of the four UPK2-positive cases with copy number gain exhibited trisomy 11, while one harbored an unbalanced translocation t(11;18)(q23.3;q12.1), accompanied by a copy number gain of the *UPK2* locus. These findings suggest that *UPK2* copy number gain may contribute to its overexpression in a subset of CRCs. In an expanded analysis of CRC-recurrent copy number variation loci, UPK2-positive tumors were also associated with 20q gain (*p* = 0.034) (Table S6).

Next, *UPK2* expression was examined in the TCGA dataset. Given the strong correlation between UPK2 protein and mRNA expression, along with its tumor cell-specific expression, bulk RNA expression was considered a reliable proxy for UPK2 status. In pan-cancer analysis, *UPK2* expression was highest in bladder tumors (Fig. S5), but a subset of CRCs also showed high expression, considerably upregulated relative to normal colorectal mucosa (Fig. S6), in contrast to many other tumor types in which there were no significant tumor-normal difference. Among the 467 TCGA CRC cases (485 for copy number analysis), 76 (16.3%) tumors (84, 17.3% for copy number analysis) were defined as *UPK2*-positive and matching other inclusion criteria (see methods) for subsequent analyses.

**Fig. 3:**
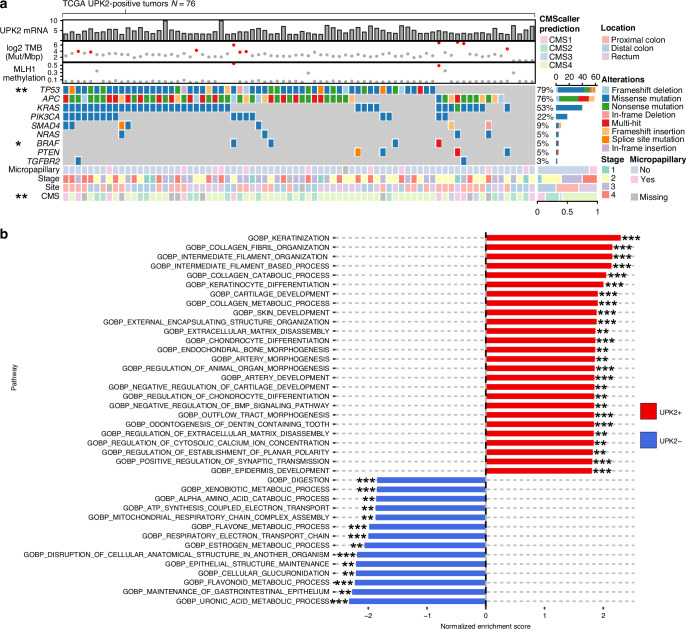
Somatic mutations and gene expression patterns in *UPK2*-positive colorectal cancer. **a** Heatmap displaying the mutation frequency of common colorectal cancer-associated mutations in *UPK2*-positive colorectal cancers from the TCGA cohort (*N* = 76), along with the basic clinicopathologic features of the tumors. **b** Gene set enrichment analysis of *UPK2*-positive (vs. other) colorectal cancer. The analyses are based on the TCGA cohort (*N*  =  467). *p value < 0.05, **p value < 0.01, ***p value < 0.001. CMS consensus molecular subtype, TMB tumor mutational burden.

The clinical and molecular features of UPK2-positive tumors in the TCGA cohort are summarized in Fig. 3a. Of the common CRC-associated mutations, *TP53* mutations were more prevalent in *UPK2*-positive CRCs (79%) compared to other cases (60%, *p* = 0.0017) in the TCGA cohort. This finding was validated in Cohorts 1 and 2, where mutation-type TP53 expression pattern was more common in UPK2-positive cases (*p* = 0.099 in Cohort 1; *p* < 0.0001 in Cohort 2) (Fig. S7). Conversely, *BRAF* mutations were less common in *UPK2*-positive CRCs (5% vs. 14%, *p* = 0.036). Morphologically, *UPK2*-positive CRCs had an overrepresentation of micropapillary CRCs [13% vs. 3%, *p* = 0.0012], consistent with observations in Cohorts 1 and 2.

To corroborate our observation of increased copy number at the UPK2 locus, we analyzed GISTIC thresholded copy number variation data and observed overrepresentation of copy number gain in the respective locus in UPK2-positive CRCs [25% vs. 12%, *p* = 0.0029]. Across 11q23.3, expression of most genes paralleled copy-number status, whereas *UPK2* displayed a weaker expression-copy number relationship (Fig. S8), suggesting that additional regulatory mechanisms contribute to *UPK2* upregulation. UPK2-positive tumors were associated with shorter progression-free interval (*p* = 0.0057) but not disease-specific survival (*p* = 0.19) in the TCGA cohort (Fig. S9). Conversely, copy-number gain encompassing the UPK2 locus at 11q23.3 showed an association with longer disease-specific survival (*p* = 0.035).

**Fig. 4:**
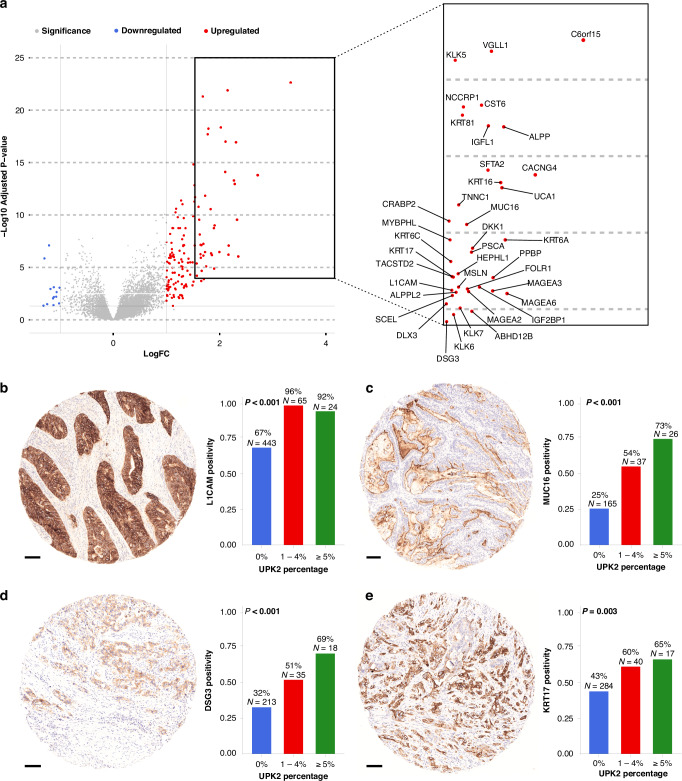
Differentially expressed genes and immunohistochemical validations in *UPK2*-positive colorectal cancer. **a** Volcano plot of the differentially expressed genes in *UPK2*-positive (vs. other) colorectal cancer in the TCGA cohort. Example tissue microarray cores of L1CAM (**b**), MUC16 (CA125) (**c**), DSG3 (**d**), and KRT17 (**e**) and corresponding bar charts showing increased expression in UPK2-positive cancers. Panel **b**–**e** are based on Cohort 1 [*N* = 760 (L1CAM and MUC16), *N* = 759 (DSG3 and KRT17)]. Scalebars, 100 μm.

In Consensus Molecular Subtype classification, *UPK2*-positive CRCs were frequently categorized as CMS4 [58% vs. 33% in *UPK2*-negative, *p* = 0.0001] (Fig. 3a and Fig. S10), a subtype known for its association with epithelial-mesenchymal transition (EMT), and less frequently categorized as CMS3. To further elucidate the biological characteristics of UPK2-positive CRCs, gene set enrichment analysis was performed (Fig. 3b). *UPK2*-positive CRCs showed enrichment for gene sets linked to keratinization, collagen metabolic process, and extracellular matrix detachment, among others. Conversely, negative enrichment scores were observed for processes related to uronic acid metabolism and maintenance of gastrointestinal epithelium, among others. A volcano plot of differentially expressed genes is shown in Fig. 4a. Expression averages for all studied genes are presented in Supplementary File 2. We selected *L1CAM, MUC16, DSG3, and KRT17* for immunohistochemical validation based on available monoclonal antibodies against these targets. All markers were primarily expressed in the tumor epithelium, and UPK2-positive CRCs showed higher expression of all selected proteins (*p* < 0.01) (Fig. 4b–e). To explore putative interaction partners, we queried the STRING database with the differentially expressed gene set [22]. UPK2 clustered with KRT6A and in a broader analysis with other keratins, as well (Fig. S11). In contrast, under the selected parameters, no significant predicted associations were observed between UPK2 and EMT-related genes. Overall, these findings suggest that UPK2-positive CRCs are characterized by gene expression profiles indicative of EMT, differentiation towards squamous epithelia, and reduced intestinal differentiation.

## Discussion

We examined the prognostic, histopathological, immunological, and molecular characteristics of UPK2-positive CRC using three independent cohorts totaling 2318 cases. UPK2 expression was significantly associated with shorter CSS and marked by a reduction in antitumorigenic immune cell infiltration, including T cells, B cells, and plasma cells. On a molecular level, UPK2-positive CRC frequently exhibited *TP53* mutation and gene expression profiles suggestive of EMT, differentiation towards squamous epithelia, and reduced intestinal differentiation. From a clinical standpoint, the most immediate impact of the results is prognostic and biological: UPK2 highlights a small, adverse-feature CRC subset that may be useful for risk stratification. To our knowledge, UPK2 is not under active therapeutic development in urothelial or other cancers. Whether UPK2-positive CRCs require modified or intensified therapy is unknown and will require treatment outcome data stratified by UPK2 status.

UPK2 expression was observed in approximately 12% of CRCs, with significant variation in expression levels across tumors. Pan-cancer analysis demonstrated that *UPK2* expression was elevated in a subset of CRCs compared to normal tissue, suggesting that UPK2 expression may delineate a distinct biological subgroup. Research on UPK2 expression in non-urothelial cancers has been limited, as it has been considered relatively specific for urothelial carcinoma [8, 26]. For instance, Hoang et al. [8] conducted an immunohistochemical study of UPK2 expression and reported no UPK2-positive colon adenocarcinomas and only one UPK2-positive prostatic adenocarcinoma among all solid cancer cases examined (*N* = 376). Similarly, Mochizuki et al. [26] observed minimal UPK2 positivity in non-urothelial cancers, with only one positive colon cancer case and a few others. Tian et al. [27] found no UPK2 expression in breast and prostate carcinomas. However, these studies were limited by small sample sizes for individual cancer types. In contrast, our study, involving a larger sample, revealed a higher frequency of UPK2 expression in CRC, highlighting the need for larger studies to better understand the role of UPK2 in colorectal and other cancers.

Although the precise role of UPK2 expression in CRC remains unclear, our findings provide a comprehensive characterization that establishes a basis for future mechanistic investigations. Insights from urothelial biology, where UPK2 contributes to barrier integrity, permeability regulation, and membrane stabilization, may inform potential functions in CRC [8]. Hypothetically, UPK2 expression could reinforce cell-surface barrier properties to limit immune infiltration, reduce passive uptake of hydrophilic chemotherapeutics, or stabilize membrane domains during partial EMT, thereby facilitating invasive behavior while retaining selected epithelial traits. These hypotheses are speculative and require experimental validation to delineate whether UPK2 is mechanistically involved in CRC pathogenesis or functions primarily as a biomarker of broader biological processes.

Optical genome mapping, a high-resolution technique for analyzing chromosomal alterations, indicated that 33% of UPK2-positive CRCs (vs. 4.3% in UPK2-negative CRCs) exhibited copy number gain involving the *UPK2* locus. Similar results were observed in the TCGA cohort. However, *UPK2* expression showed a weaker dependence on copy number than many other genes in 11q23.3 locus, suggesting that additional mechanisms, such as epigenetic regulation or transcriptional activation, may contribute to UPK2 expression and warrant further exploration.

UPK2 positivity was linked to more aggressive cancer characteristics, including higher tumor grade, advanced disease stage, nodal and distant metastasis, lymphovascular invasion, micropapillary growth pattern, and tumor budding. Consistent with these findings, survival analyses revealed that UPK2 expression was associated with shorter CSS in both cohorts, with this finding remaining significant in multivariable analysis in Cohort 2. Differences in baseline characteristics, institutional practice, and calendar-time changes in management may contribute to between-cohort variation in survival estimates, and residual selection bias cannot be excluded. To date, there have been no other studies focusing on the prognostic role of UPK2 in CRC. However, studies in other cancers have yielded mixed results. For instance, high plasma *UPK2* mRNA levels were linked to shorter overall survival in lung adenocarcinoma [28], whereas UPK2 expression in urothelial carcinomas showed no significant association with recurrence-free or overall survival [29]. Future studies could investigate whether *UPK2* expression may serve as a useful marker for identifying high-risk stage II colorectal cancers that could benefit from adjuvant therapy.

Microsatellite instability or MMR deficiency is an important biological feature in CRC associated with better prognosis and implications for adjuvant therapies, particularly immune checkpoint inhibitors and chemotherapy effectiveness [30, 31]. In this study, UPK2 expression was predominantly observed in MMR proficient tumors, which are typically less immunologically active. Even when restricting the analysis to MMR proficient tumors, UPK2-positive CRCs demonstrated reduced densities of antitumorigenic immune cells, including CD3^+^ T cells, CD20^+^CD79A^+^ B cells, and CD20^-^CD79A^+^ plasma cells, as revealed through multiplex immunohistochemistry. Histological assessment of H&E-stained slides also indicated weaker lymphocytic reactions in these tumors. To our knowledge, no prior studies have investigated the relationships between UPK2 expression and tumor immune microenvironment. Future research should focus on elucidating the mechanisms behind the diminished lymphocytic response observed in UPK2-positive CRCs and its potential implications for immunotherapy resistance.

Beyond the MMR proficient phenotype, UPK2-positive CRCs frequently harbored *TP53* mutations, which are known to disrupt tumor suppressor functions and promote carcinogenesis [32]. *UPK2* is not listed among experimentally supported TP53 targets in a comprehensive survey [33], and our STRING-based association network did not reveal strong links between UPK2 and canonical TP53-pathway genes. Although the prognostic significance of *TP53* mutation in CRC remains debated, with some studies indicating poorer survival outcomes [34] and others reporting no significant impact [35], our results suggest a potential role for *TP53* mutation in the phenotypically aggressive UPK2-positive tumors, which may be mediated through EMT activation [36, 37]. Additionally, gene expression profiling revealed enrichment of genes associated with keratinization and differentiation towards squamous epithelia, such as *KRT17*, *KRT6A*, *KRT4*, *KRT81, and DSG3*. While various keratins have traditionally been used as tissue type-specific epithelial markers in cancer diagnostics, some of them have also been implicated in tumor invasiveness and poor prognosis [38–40]. The enrichment of keratinization and increased expression of basal cytokeratins, including KRT17 and KRT6, in UPK2-positive tumors are reminiscent of the basal/quasi-mesenchymal subtype of pancreatic ductal adenocarcinoma [41–43], which has been associated with worse prognosis [44]. UPK2-positive CRCs were also enriched for CMS4 and negatively enriched for CMS3. Notably, CMS4 is heterogeneous; the present data suggest that a subset may exhibit prominent basal-like epithelial characteristics rather than a purely mesenchymal/stromal program. In the STRING network analysis, UPK2 also clustered with keratins, aligning with the basal-like pattern. Together, these findings raise a hypothesis of a potentially unrecognized CMS4 subgroup that is identified by basal-like features. Pathways associated with EMT, a process by which epithelial cells acquire mesenchymal properties, facilitating tumor cell spread [45], were also enriched in UPK2-positive CRCs. Immunohistochemical validation confirmed higher expression of EMT-associated markers L1CAM and MUC16 in these tumors. Furthermore, UPK2-positive CRCs were predominantly classified within Consensus Molecular Subtype 4, characterized by EMT activation, extracellular matrix remodeling, and a poor prognosis [46, 47]. Protein-protein association analysis, however, revealed no apparent interactions between UPK2 and EMT-related genes, suggesting that these features may arise from broader tumor programs rather than direct UPK2-EMT linkages. Overall, these molecular traits further highlight the aggressive nature of UPK2-positive CRCs.

Several limitations should be acknowledged. The assessment of UPK2 expression was subjective. However, strong interobserver agreement and consistent expression rates across cohorts enhance the reliability of these findings. The use of mRNA in situ hybridization for orthogonal validation strengthens the robustness of immunohistochemistry results. The present study does not extend to mechanistic interrogation of UPK2. Future work could include UPK2 loss- and gain-of-function studies across molecular subtypes, employing xenograft or organoid models to determine whether UPK2 modulation alters tumor growth, phenotype, or lineage programs. Our protein-protein association findings were based on the STRING database, and do not necessarily establish direct biochemical interactions. These findings merit verification with other biochemical, biophysical, or genetic approaches. Exclusion of neoadjuvant-treated patients limits the generalizability of findings to this subgroup. Additionally, both UPK2 expression and immune cell densities were assessed using tissue microarrays, which may overlook intratumor heterogeneity. However, tissue microarrays are an established method for investigating tumor biomarkers and the immune landscape, enabling analyses of large cohorts. While scoring reproducibility was high (κ = 0.75), the prognostic significance of UPK2 may be influenced by cohort-specific factors, including differences in postoperative mortality and patient characteristics. Further validation in independent, balanced cohorts is warranted.

The strengths of this study include its large sample size, multi-cohort design, and comprehensive methodological approach. The inclusion of over 1800 cases with detailed clinicopathologic annotations enabled robust multivariable survival analyses. Multiplex immunohistochemistry provided more detailed analyses of immune cells than is possible with conventional single-plex methods. Furthermore, the TCGA cohort and optical genome mapping offered precise molecular characterization of UPK2-positive CRCs, and the main findings were validated across independent datasets.

In conclusion, UPK2 is expressed in a subset of CRCs characterized by aggressive histomorphological characteristics, poor survival outcomes, and distinct molecular and immunological features. These findings advance the understanding of CRC heterogeneity and suggest that UPK2 could serve as a novel prognostic marker. Future studies should further investigate the biological mechanisms underlying UPK2 expression and its potential role in guiding personalized treatment strategies for CRC.

## Supplementary information

REMARK checklist

Supplementary material

Supplementary File 1

Supplementary File 2

## Data Availability

Data generated and/or analyzed during this study are not publicly available. The sharing of data will require approval from relevant ethics committees and/or biobanks. Further information including the procedures to obtain and access data of Finnish Biobanks are described at https://finbb.fi/en/fingenious-service.
