# Supplementary material for: Urothelium marker UPK2 identifies aggressive colorectal cancers with distinct molecular and histological features
Source: Br J Cancer. 2025 Dec 11;134(4):650–61. doi: 10.1038/s41416-025-03300-1 (PMC12858874; doi:10.1038/s41416-025-03300-1)
Supplement: Supplementary file 2 — Supplementary material [file 41416_2025_3300_MOESM2_ESM.pdf]

Supplementary tables

Table S1. Comparison of differences in tumor and patient characteristics between Cohorts 1 and 2.

Characteristic	Cohort 1	Cohort 2	p value
All cases	761 (100%)	1090 (100%)	
Sex			0.31
Female	358 (47%)	539 (49%)	
Male	403 (53%)	551 (51%)	
Age (years)			0.030
<65	229 (30%)	285 (26%)	
65-75	280 (37%)	381 (35%)	
>75	252 (33%)	424 (39%)	
Year of operation			<0.0001
2000-2005	-	339 (31%)	
2006-2010	151 (20%)	351 (32%)	
2011-2015	212 (28%)	400 (37%)	
2016-2020	398 (52%)	-	
Tumor location			<0.0001
Proximal colon	319 (42%)	530 (49%)	
Distal colon	204 (27%)	402 (37%)	
Rectum	238 (31%)	158 (14%)	
WHO grade			0.0039
Low	651 (86%)	876 (80%)	
High	110 (14%)	214 (20%)	
UICC disease stage			0.0028
I	175 (23%)	182 (17%)	
II	252 (33%)	406 (37%)	
III	250 (33%)	352 (32%)	
IV	84 (11%)	150 (14%)	
T			0.0001
T1-T2	216 (28%)	223 (20%)	
T3-T4	545 (72%)	867 (80%)	
N			0.68
N0	441 (58%)	621 (57%)	
N1-N2	320 (42%)	469 (43%)	
M			0.083
M0	677 (89%)	940 (86%)	
M1	84 (11%)	150 (14%)	
Lymphovascular invasion			<0.0001
No	416 (55%)	850 (78%)	
Yes	345 (45%)	240 (22%)	
Micropapillary growth pattern			0.096
0%	693 (91%)	993 (91%)	
1-4%	30 (3.9%)	27 (2.5%)	
≥5%	38 (5.0%)	70 (6.4%)	
Tumor budding			0.024
Grade 1 (0-4)	528 (69%)	818 (75%)	
Grade 2 (5-9)	128 (17%)	155 (14%)	
Grade 3 (≥10)	105 (14%)	117 (11%)	
MMR status			0.56
Proficient	639 (84%)	926 (85%)	
Deficient	122 (16%)	164 (15%)	
BRAF status[A]			0.15
Wild-type	654 (86%)	908 (83%)	
Mutant	107 (14%)	180 (17%)	

Table S2. Multivariable Cox proportional hazards regression models for cancer-specific survival and overall survival in Cohorts 1 and 2.

	Cohort 1		Cohort 2	
	Cancer-specific survival	Overall survival	Cancer-specific survival	Overall survival
	Multivariable HR (95% CI)	Multivariable HR (95% CI)	Multivariable HR (95% CI)	Multivariable HR (95% CI)
UPK2				
0%	1 (referent)	1 (referent)	1 (referent)	1 (referent)
1–4%	0.63 (0.37-1.06)	0.55 (0.35-0.89)	1.63 (1.15-2.31)	1.54 (1.17-2.03)
≥5%	1.28 (0.64-2.56)	0.85 (0.45-1.63)	2.31 (1.46-3.65)	1.83 (1.25-2.68)
Age				
<65	1 (referent)	1 (referent)	1 (referent)	1 (referent)
65–75	1.62 (1.07-2.44)	1.65 (1.16-2.34)	1.09 (0.81-1.46)	1.27 (0.99-1.63)
>75	2.44 (1.59-3.73)	4.12 (2.96-5.75)	1.76 (1.32-2.37)	2.87 (2.28-3.62)
Sex				
Male	1 (referent)	1 (referent)	1 (referent)	1 (referent)
Female	1.04 (0.75-1.45)	0.85 (0.66-1.08)	0.87 (0.68-1.11)	0.75 (0.63-0.90)
Year of operation				
2000–2005	–	–	1 (referent)	1 (referent)
2006–2010	1 (referent)	1 (referent)	0.61 (0.46-0.81)	0.69 (0.56-0.85)
2011–2015	0.82 (0.55-1.22)	0.82 (0.61-1.11)	0.48 (0.36-0.64)	0.61 (0.49-0.76)
2016–2020	0.57 (0.37-0.86)	0.67 (0.49-0.92)	–	–
WHO grade				
Low	1 (referent)	1 (referent)	1 (referent)	1 (referent)
High	1.42 (0.93-2.17)	1.21 (0.86-1.72)	1.44 (1.07-1.93)	1.58 (1.26-1.98)
Tumor location				
Proximal colon	1 (referent)	1 (referent)	1 (referent)	1 (referent)
Distal colon	1.12 (0.74-1.71)	1.05 (0.76-1.44)	0.89 (0.68-1.16)	0.96 (0.79-1.17)
Rectum	0.94 (0.62-2.17)	0.99 (0.72-1.35)	0.84 (0.58-1.21)	0.90 (0.68-1.19)
UICC disease stage				
I–II	1 (referent)	1 (referent)	1 (referent)	1 (referent)
III	2.77 (1.69-4.53)	1.29 (0.95-1.74)	3.19 (2.32-4.38)	1.53 (1.25-1.89)
IV	20.3 (12.0-34.4)	8.50 (5.92-12.2)	18.2 (13.0-25.6)	8.01 (6.26-10.3)
Lymphovascular invasion				
No	1 (referent)	1 (referent)	1 (referent)	1 (referent)
Yes	2.14 (1.38-3.31)	1.44 (1.08-1.90)	1.88 (1.46-2.43)	1.53 (1.25-1.88)
MMR status				
MMR proficient	1 (referent)	1 (referent)	1 (referent)	1 (referent)
MMR deficient	0.45 (0.22-0.94)	0.87 (0.57-1.34)	0.67 (0.39-1.12)	0.77 (0.54-1.10)
BRAF mutation				
Wild-type	1 (referent)	1 (referent)	1 (referent)	1 (referent)
Mutant	1.61 (0.91-2.86)	1.34 (0.89-2.03)	1.24 (0.81-1.91)	1.39 (1.02-1.91)

Abbreviations: UICC, Union for International Cancer Control; MMR, Mismatch repair

Table S3. Comparison of the prognostic power of UPK2 expression and tumor budding using Cox regression models for cancer-specific survival.

Variable	No. of cases	No. of events	Model 1 (Univariable) HR (95% CI)	Model 2 (multivariable) HR (95% CI)	Model 3 (multivariable) HR (95% CI)
**Cohort 1**					
UPK2 status					
0%	662	126	1 (referent)	1 (referent)	1 (referent)
1-4%	68	18	1.42 (0.87-2.32)	0.93 (0.56-1.55)	0.54 (0.32-0.93)
≥5%	26	9	1.97 (1.00-3.88)	1.41 (0.71-2.79)	1.15 (0.57-2.33)
P_{trend}			0.021	0.56	0.29
Tumor budding					
0-4	538	76	1 (referent)	1 (referent)	1 (referent)
5-9	127	35	2.13 (1.43-3.18)	2.15 (1.44-3.23)	1.67 (1.08-2.57)
≥10	106	44	3.68 (2.54-5.34)	3.74 (2.53-5.51)	2.18 (1.44-3.31)
P_{trend}			<0.0001	<0.0001	0.0002
**Cohort 2**					
UPK2 status					
0%	918	227	1 (referent)	1 (referent)	1 (referent)
1-4%	96	44	2.29 (1.66-3.16)	1.79 (1.29-2.49)	1.55 (1.08-2.21)
≥5%	39	22	3.33 (2.15-5.16)	3.16 (2.04-4.90)	2.30 (1.54-3.64)
P_{trend}			<0.0001	<0.0001	<0.0001
Tumor budding					
0-4	803	175	1 (referent)	1 (referent)	1 (referent)
5-9	148	61	2.24 (1.68-3.00)	2.08 (1.55-2.81)	1.29 (0.89-1.69)
≥10	112	60	3.20 (2.39-4.30)	2.92 (2.17-3.95)	1.32 (0.96-1.82)
P_{trend}			<0.0001	<0.0001	0.071

Model 2: Cox proportional hazards regression model that included UPK2 status and tumor budding.

Model 3: Cox proportional hazards regression model that included UPK2 status and tumor budding and was additionally adjusted for age (<65, 65-75, <75), sex (male, female), stage (I-II, III, IV), tumor location (proximal colon, distal colon, rectum), year of operation (2000-2005, 2006-2010, 2011-2015), lymphovascular invasion (no, yes), grade (low-grade, high-grade), MMR status (proficient, deficient) and BRAF (wild-type, mutant).
Abbreviations: HR, hazard ratio; CI, confidence interval

Table S4. Associations of lymphocytic reaction patterns with UPK2 expression in mismatch-repair proficient tumors in Cohort 2 (N = 1,100).

Characteristic	Total	UPK2			p value
		0%	1–4%	≥5%	
Peritumoral reaction					0.016
Absent	163 (18%)	131 (80%)	20 (12%)	12 (7.4%)	
Low	379 (41%)	318 (84%)	43 (11%)	18 (4.7%)	
Intermediate	327 (35%)	285 (87%)	34 (10%)	8 (2.4%)	
High	57 (6.2%)	56 (98%)	1 (1.8%)	0 (0.0%)	
Intratumoral periglandular reaction					0.005
Absent	161 (17%)	126 (78%)	23 (14%)	12 (7.5%)	
Low	366 (40%)	305 (83%)	42 (11%)	19 (5.2%)	
Intermediate	367 (40%)	328 (89%)	32 (8.7%)	7 (1.9%)	
High	32 (3.5%)	328 (89%)	32 (8.7%)	7 (1.9%)	
Tumor-infiltrating lymphocytes					0.014
Absent	598 (65%)	494 (83%)	70 (12%)	34 (5.7%)	
Low	256 (28%)	227 (89%)	25 (9.8%)	4 (1.6%)	
Intermediate	63 (6.8%)	60 (95%)	3 (4.8%)	0 (0.0%)	
High	9 (1.0%)	9 (100%)	0 (0.0%)	0 (0.0%)	
Crohn’s-like lymphoid reaction					0.051
Absent	303 (33%)	246 (81%)	37 (12%)	20 (6.6%)	
Low	390 (42%)	335 (86%)	42 (11%)	13 (3.3%)	
Intermediate	174 (19%)	157 (90%)	12 (6.9%)	5 (2.9%)	
High	59 (6.4%)	52 (88%)	7 (12%)	0 (0.0%)	

Table S5. Immune cell density associations with UPK2 status in Cohort 1.

Immune cell type	N	UPK2 0%	UPK2 1–4%	UPK2 ≥5%	p
**Tumor intraepithelial region**					
CD3+ T cells	638	99 (46–193)	62 (29–135)	51 (27–117)	0.0003
CD8+ T cells	638	71 (26–154)	44 (15–100)	36 (14–118)	0.0032
$CD20^+CD79A^+$ B cells	638	0 (0–0)	0 (0–0)	0 (0–1)	0.24
$CD20^-CD79A^+$ plasma cells	638	0 (0–0)	0 (0–0)	0 (0–0)	0.65
**Tumor stromal region**					
CD3+ T cells	638	1,136 (789–1,594)	987 (591–1,406)	923 (544–1,273)	0.013
CD8+ T cells	638	404 (240–696)	301 (164–514)	304 (127–593)	0.0071
$CD20^+CD79A^+$ B cells	638	30 (8–78)	30 (8–54)	20 (9–78)	0.79
$CD20^-CD79A^+$ plasma cells	638	113 (34–246)	55 (17–213)	56 (27–142)	0.0095

Table S6. Common colorectal cancer associated chromosome copy number alterations in UPK2-positive and negative colorectal cancers evaluated using optical genome mapping (N=35).

	UPK2-negative	UPK2-positive	P
8p loss	7/23 (30 %)	5/12 (42 %)	0.71
8q gain	11/23 (48 %)	9/12 (75 %)	0.16
13q gain	9/23 (39 %)	8/12 (67 %)	0.16
15q loss	6/23 (26 %)	2/12 (17 %)	0.69
17p loss	9/23 (39 %)	5/12 (42 %)	>0.99
18q loss	10/23 (43 %)	4/12 (33 %)	0.72
20q gain	10/23 (43 %)	10/12 (83 %)	0.034

Supplementary figures

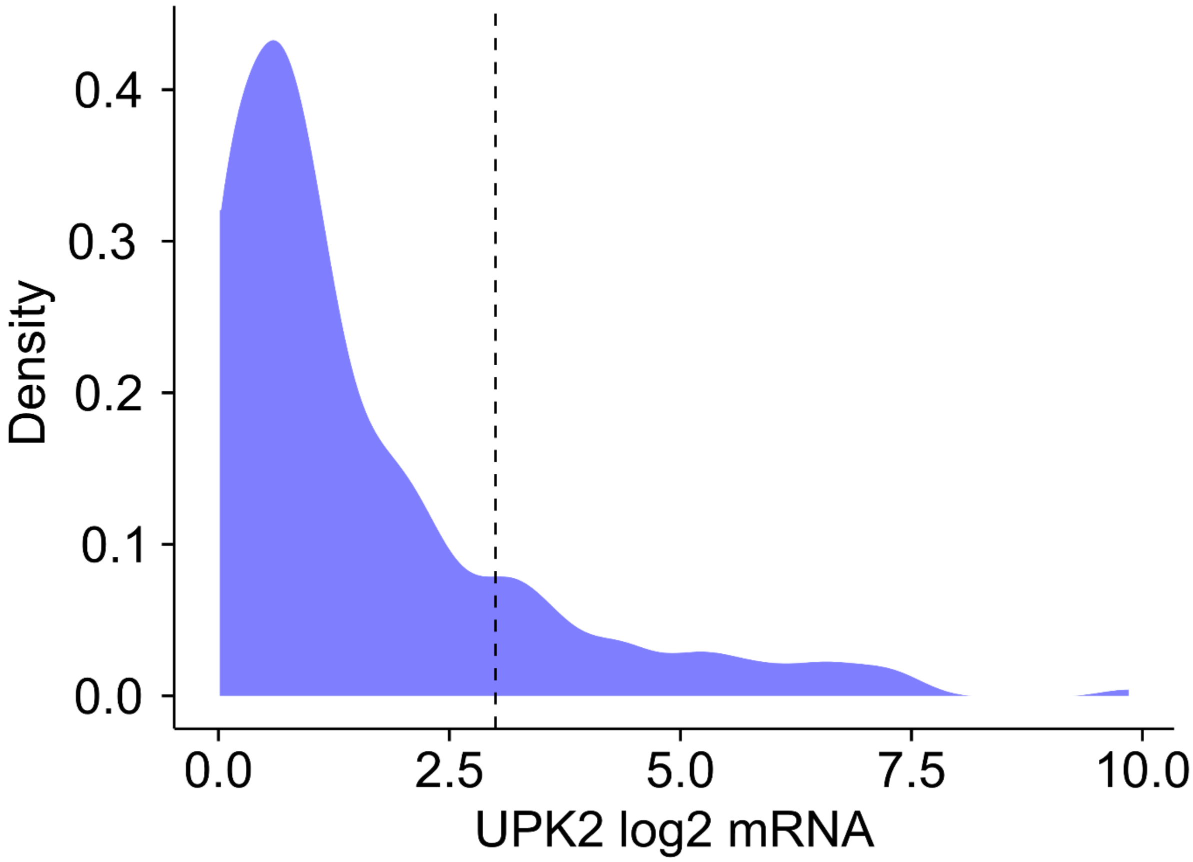

Figure S1. Distribution of *UPK2* mRNA levels in the TCGA cohort and cutoff for UPK2 positivity.

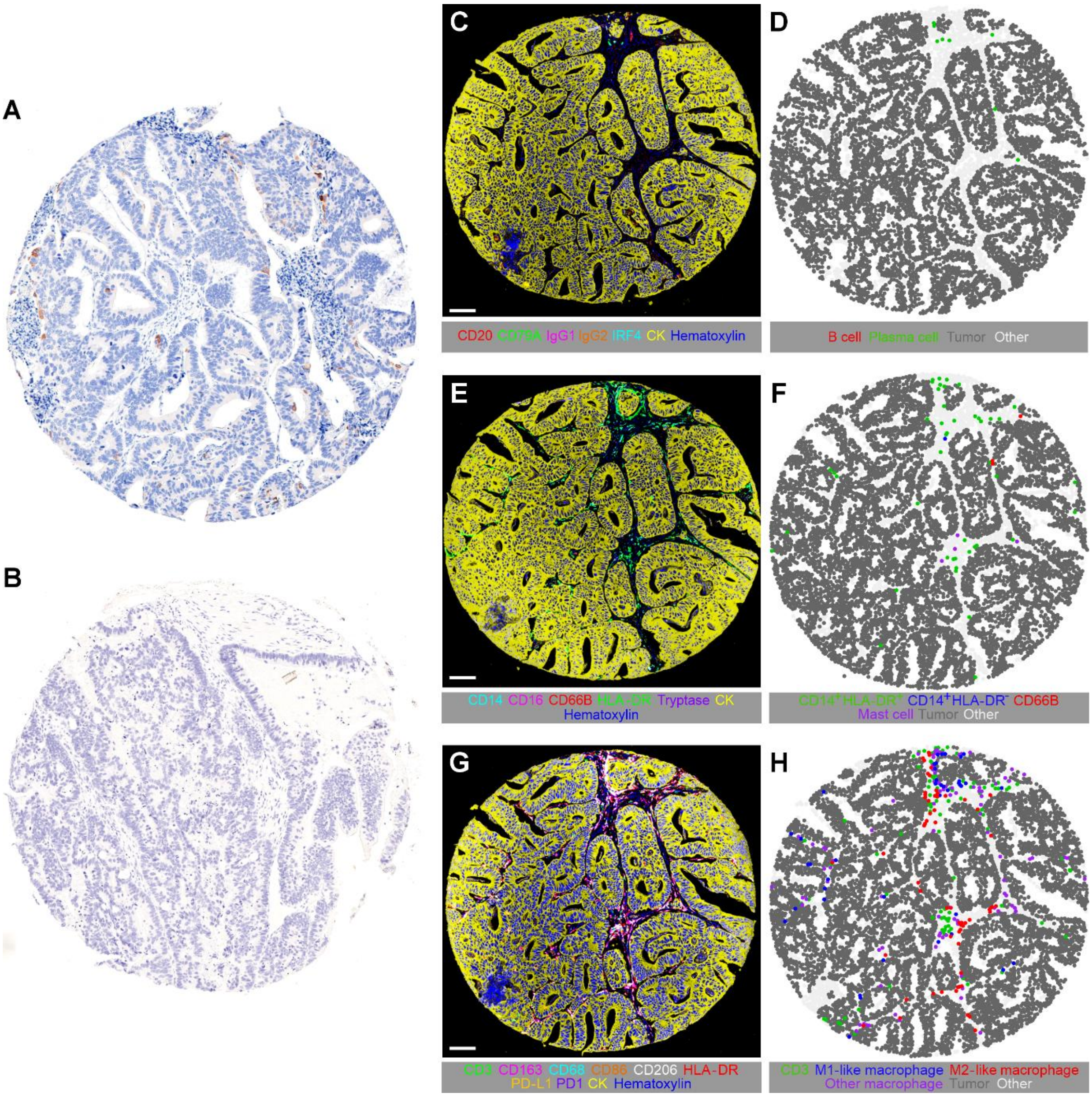

Figure S2. UPK2 staining of the example core in Figure 2 as well as another multiplex immunohistochemistry example tumor. (A) The UPK2 staining of the example core from Figure S2 displays low UPK2 expression. (B) UPK2 staining of the example multiplex immunohistochemistry core from Figure 2 displays no UPK2 expression. (C-H) Example multiplex immunohistochemistry images of a tumor (C, E, F) along with their corresponding cell maps (D, F, H), generated through machine learning-assisted image analysis.

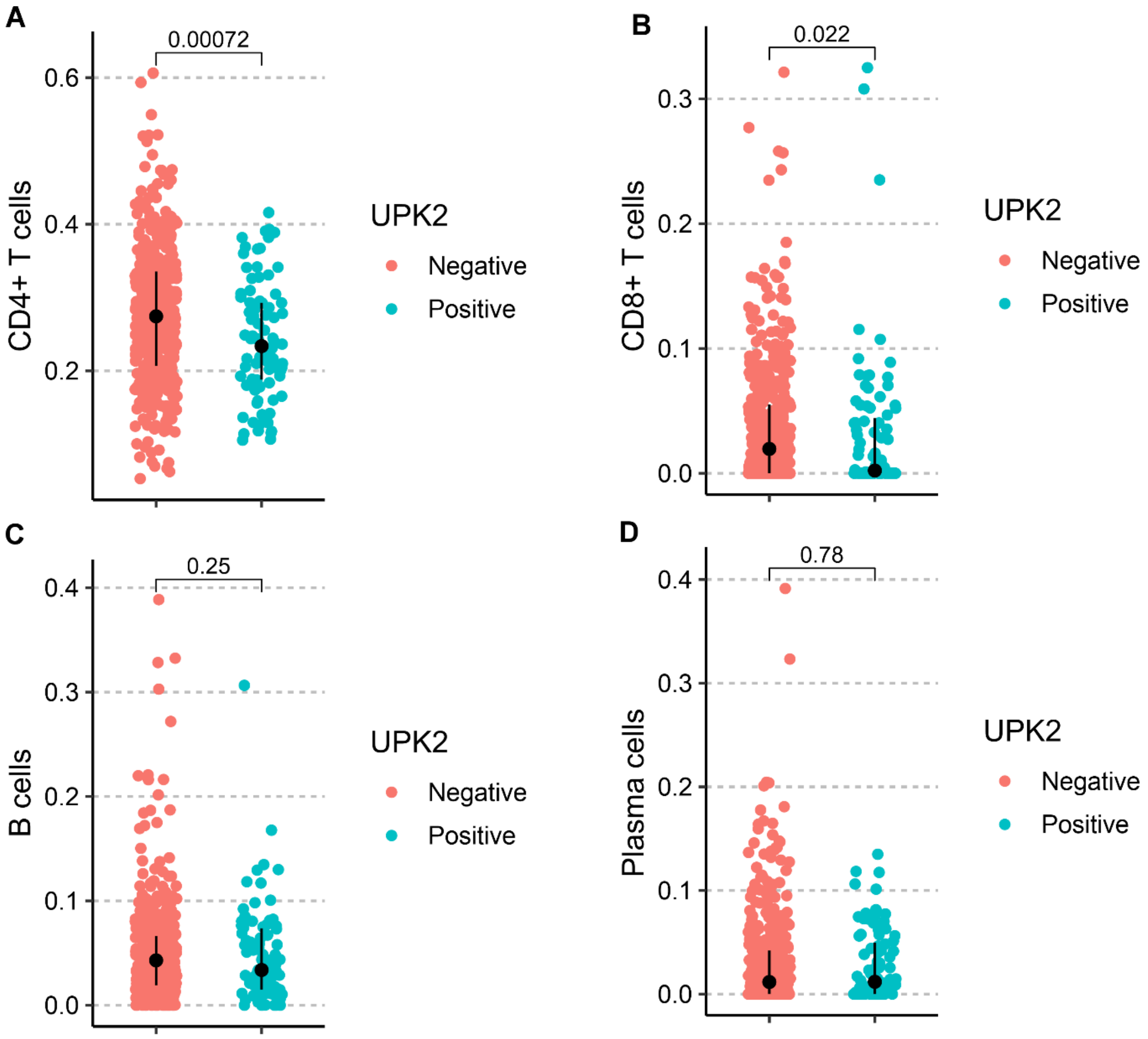

Figure S3. Lymphocytic infiltration in UPK2-positive colorectal cancers from the TCGA cohort according to CIBERSORT. Analyses show a statistically significant decrease in UPK2-positive CRCs for CD4+ T cells (A), and CD8+ T cells (B) but not for B cells (C) or Plasma cells (D). Black dots and lines represent the median and 25th and 75th quartiles.

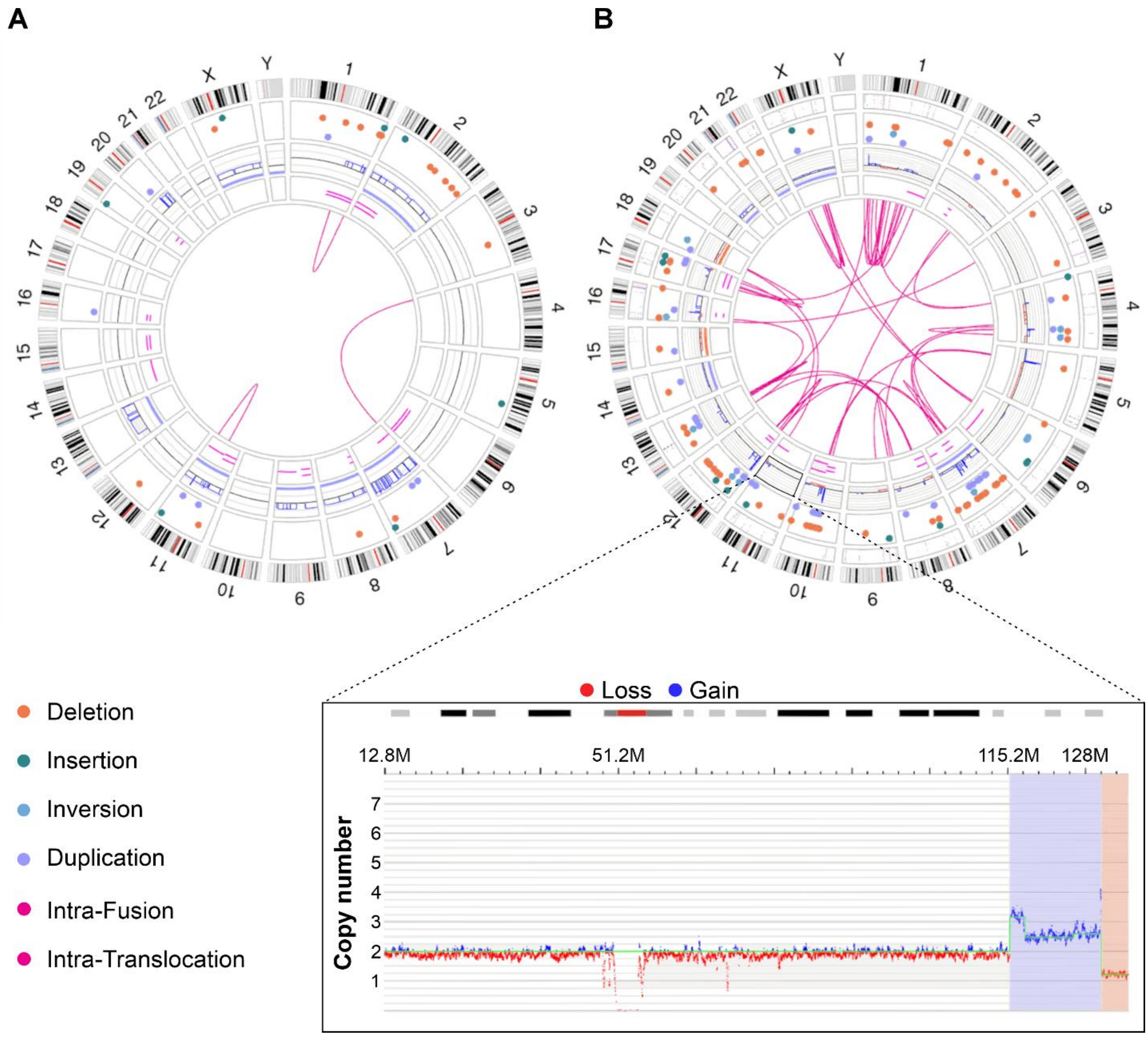

Figure S4. Optical genome mapping in UPK2-positive colorectal cancer. (A) Circos plot of a UPK2-positive colorectal cancer demonstrating trisomy of chromosome 11. (B) Circos plot a UPK2-positive colorectal cancer with t(11;18)(q23.3;q12.1) translocation and copy number gain of the *UPK2* locus.

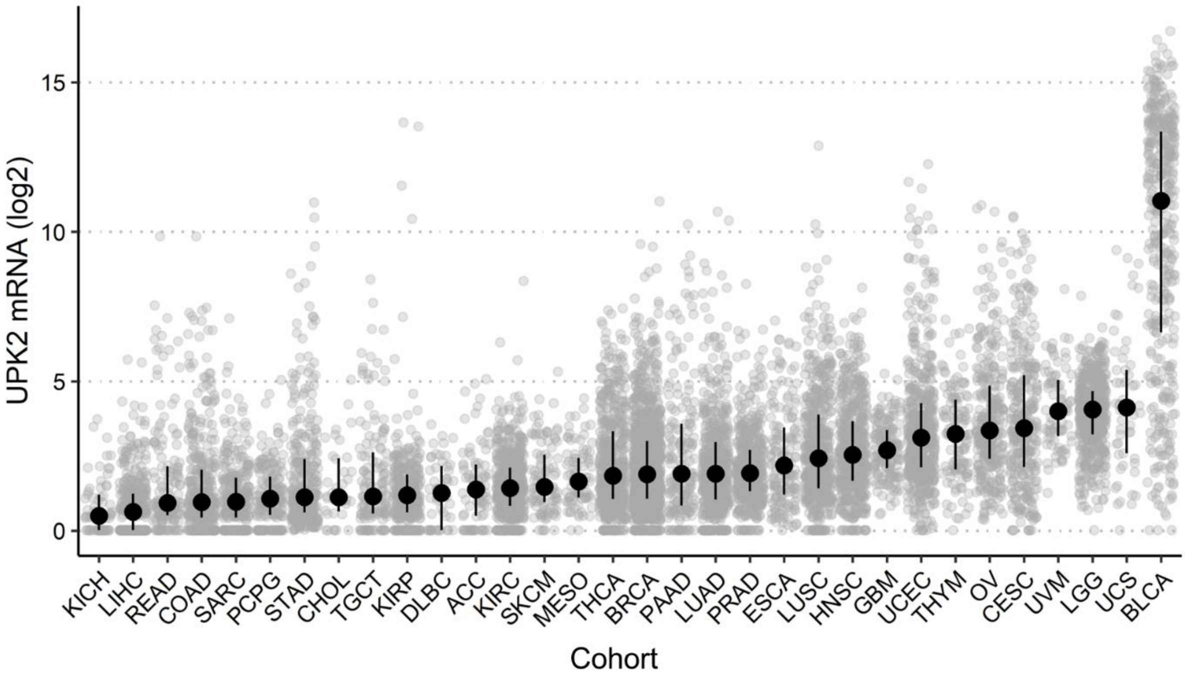

Figure S5. Pan-cancer analysis of *UPK2* mRNA expression. Bladder cancer shows the highest *UPK2* mRNA expression on average. However, various other tumor types show individual cases with high *UPK2* expression. Abbreviations: KICH, Kidney Chromophobe; LIHC, Liver Hepatocellular Carcinoma; READ, Rectal Adenocarcinoma; COAD, Colorectal Adenocarcinoma; SARC, Sarcoma; PCPG, Pheochromocytoma and Paraganglioma; STAD, Stomach Adenocarcinoma; CHOL, Cholangiocarcinoma; TGCT, Testicular Germ Cell Tumors; KIRP, Kidney renal papillary cell carcinoma; DLBC, Lymphoid Neoplasm Diffuse Large B-cell Lymphoma; ACC, Adrenocortical Carcinoma; KIRC, Kidney Renal Clear Cell Carcinoma; SKCM, Skin Cutaneous Melanoma; MESO, Mesothelioma; THCA, Thyroid Carcinoma; BRCA, Breast Carcinoma; PAAD, Pancreatic Adenocarcinoma; LUAD, Lung Adenocarcinoma; PRAD, Prostate Adenocarcinoma; ESCA, Esophageal Carcinoma; LUSC, Lung Squamous Cell Carcinoma; HNSC, Head and Neck Squamous Cell Carcinoma; GBM, Glioblastoma Multiforme; UCEC, Uterine Corpus Endometrial Carcinoma; THYM, Thymoma; OV, Ovarian Serous Cystadenocarcinoma; CESC, Cervical Squamous Cell Carcinoma and Endocervical Adenocarcinoma; UVM, Uveal Melanoma; LGG, Brain Lower Grade Glioma; UCS, Uterine Carcinosarcoma; BLCA, Bladder Urothelial Carcinoma.

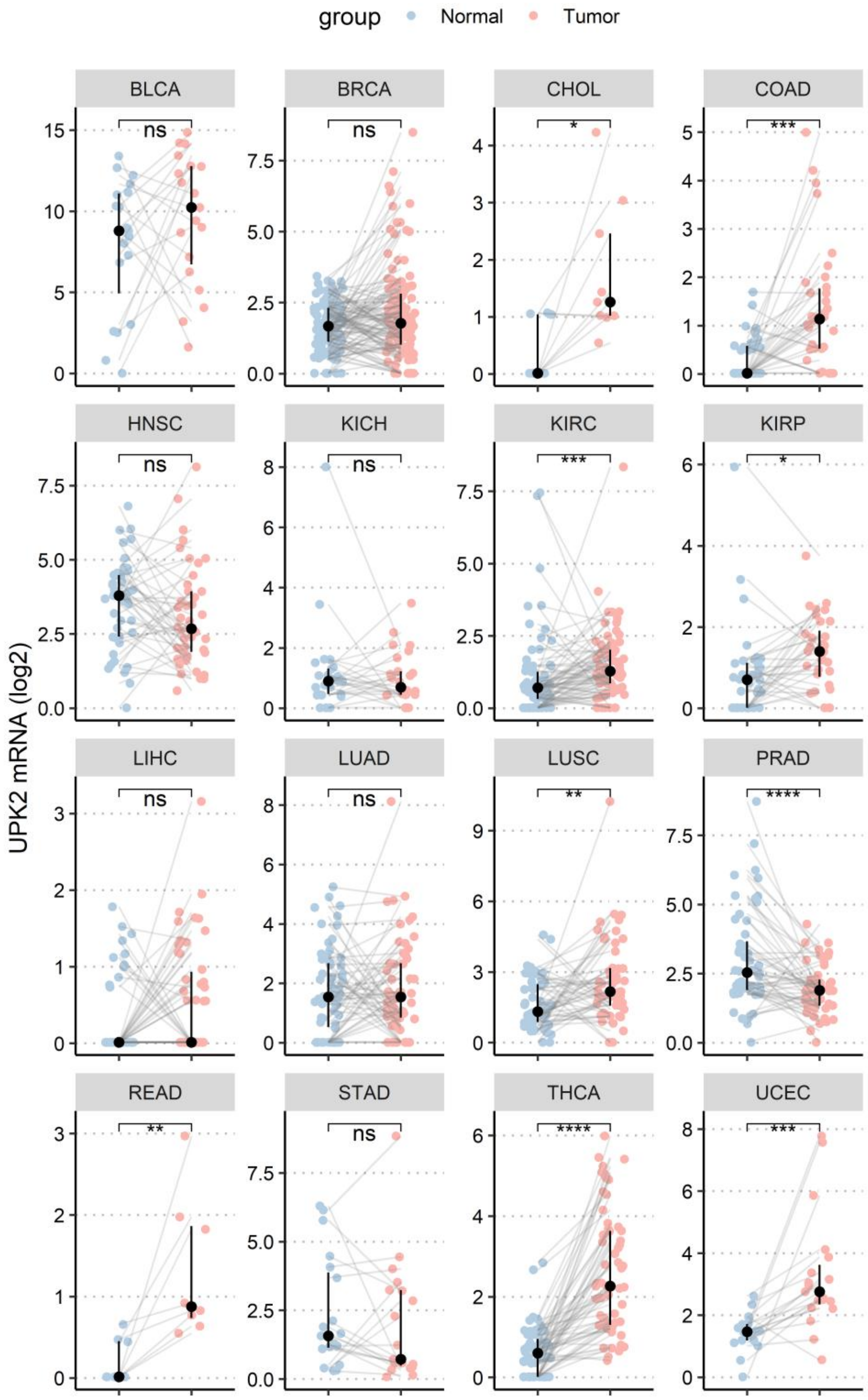

Figure S6. Pan-cancer analysis of *UPK2* mRNA expression comparing tumor and normal tissue. Both colon and rectal cancer demonstrate significantly higher levels of *UPK2* mRNA expression compared to their corresponding noncancerous mucosa. *p<0.05, **p<0.01, ***p<0.001, ****p<0.0001. Abbreviations: BLCA, Bladder Urothelial Carcinoma; BRCA, Breast Carcinoma, CHOL, Cholangiocarcinoma; COAD, Colon Adenocarcinoma; HNSC, Head and Neck Squamous Cell Carcinoma; KICH, Kidney Chromophobe; KIRC, Kidney Renal Clear Cell Carcinoma; KIRP, Kidney renal papillary cell carcinoma; LIHC, Liver Hepatocellular Carcinoma; LUAD, Lung Adenocarcinoma; LUSC, Lung Squamous Cell Carcinoma; PRAD, Prostate Adenocarcinoma; READ, Rectal Adenocarcinoma; STAD, Stomach Adenocarcinoma; THCA, Thyroid Carcinoma; UCEC, Uterine Corpus Endometrial Carcinoma.

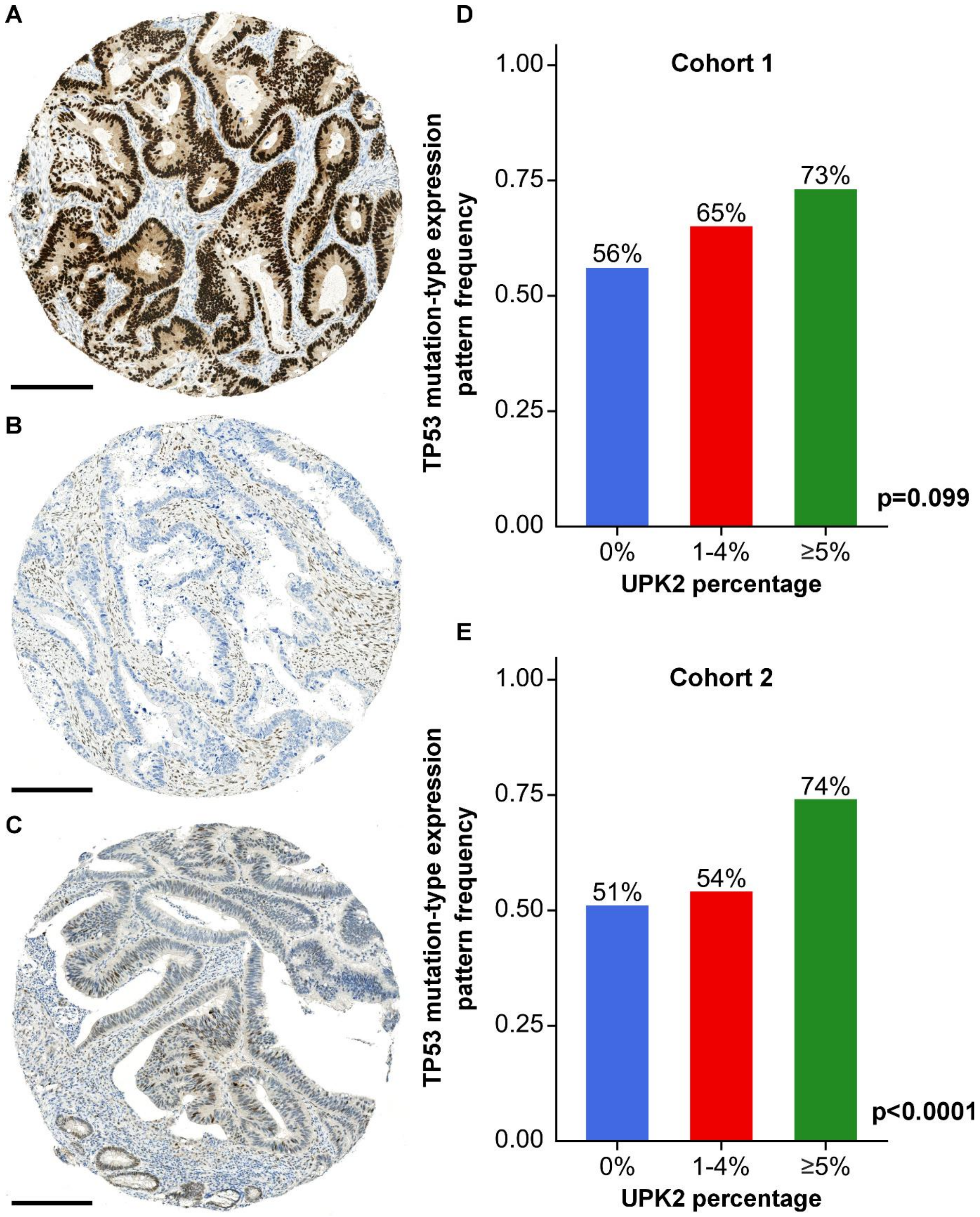

Figure S7. TP53 alterations in UPK2-positive colorectal cancer. (A and B) Tissue microarray cores of mutation-type TP53 demonstrating diffuse overexpression (A) and absent expression (B). (C) Tissue microarray core of wild-type TP53 with heterogeneous expression. (D and E) Barplots of TP53 alteration frequency according to UPK2 positivity in Cohort 1 (D) and Cohort 2 (E). Scalebar, 250µm.

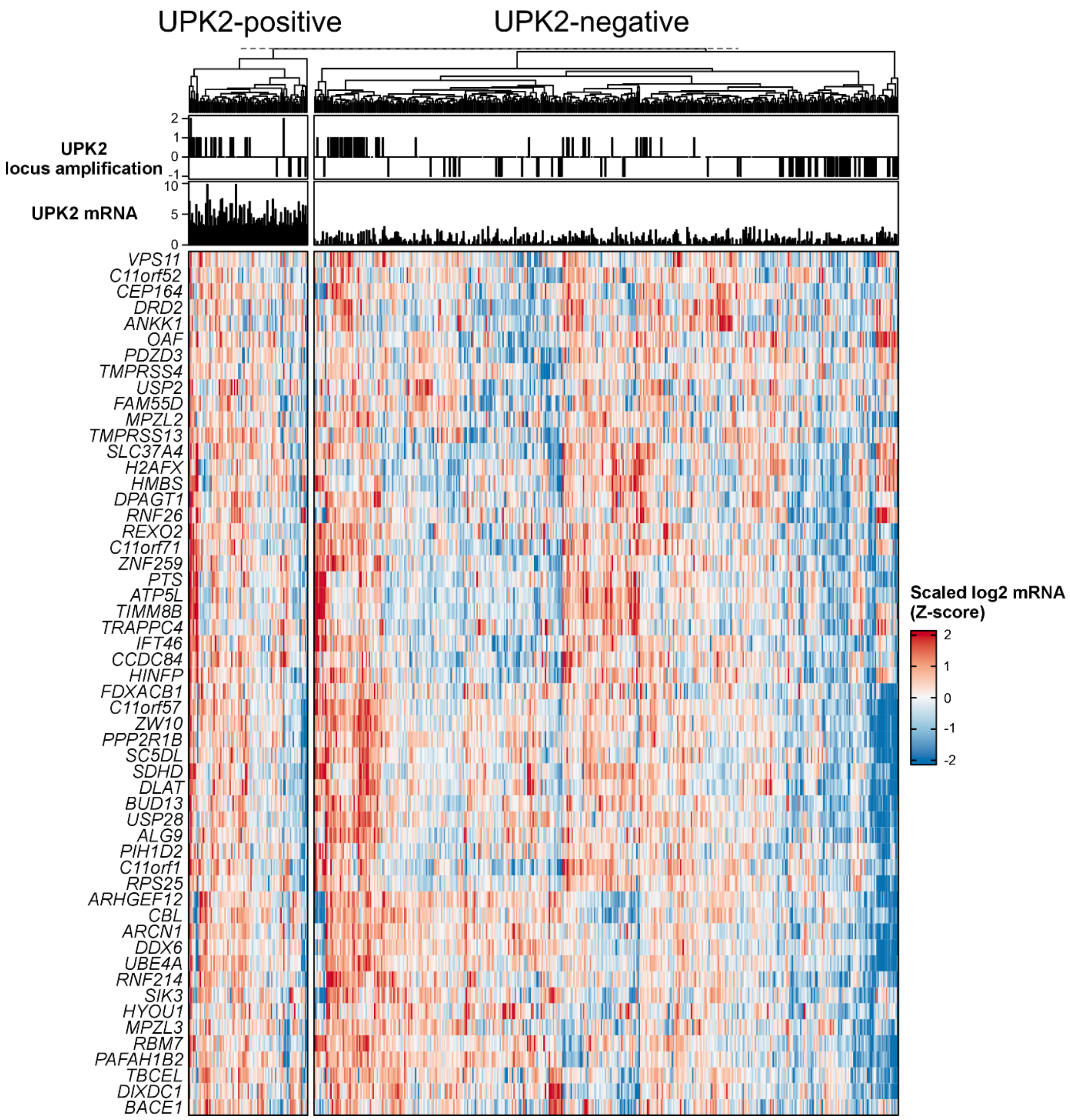

Figure S8. Heatmap of gene expressions in the *UPK2* (11q23.3) locus.

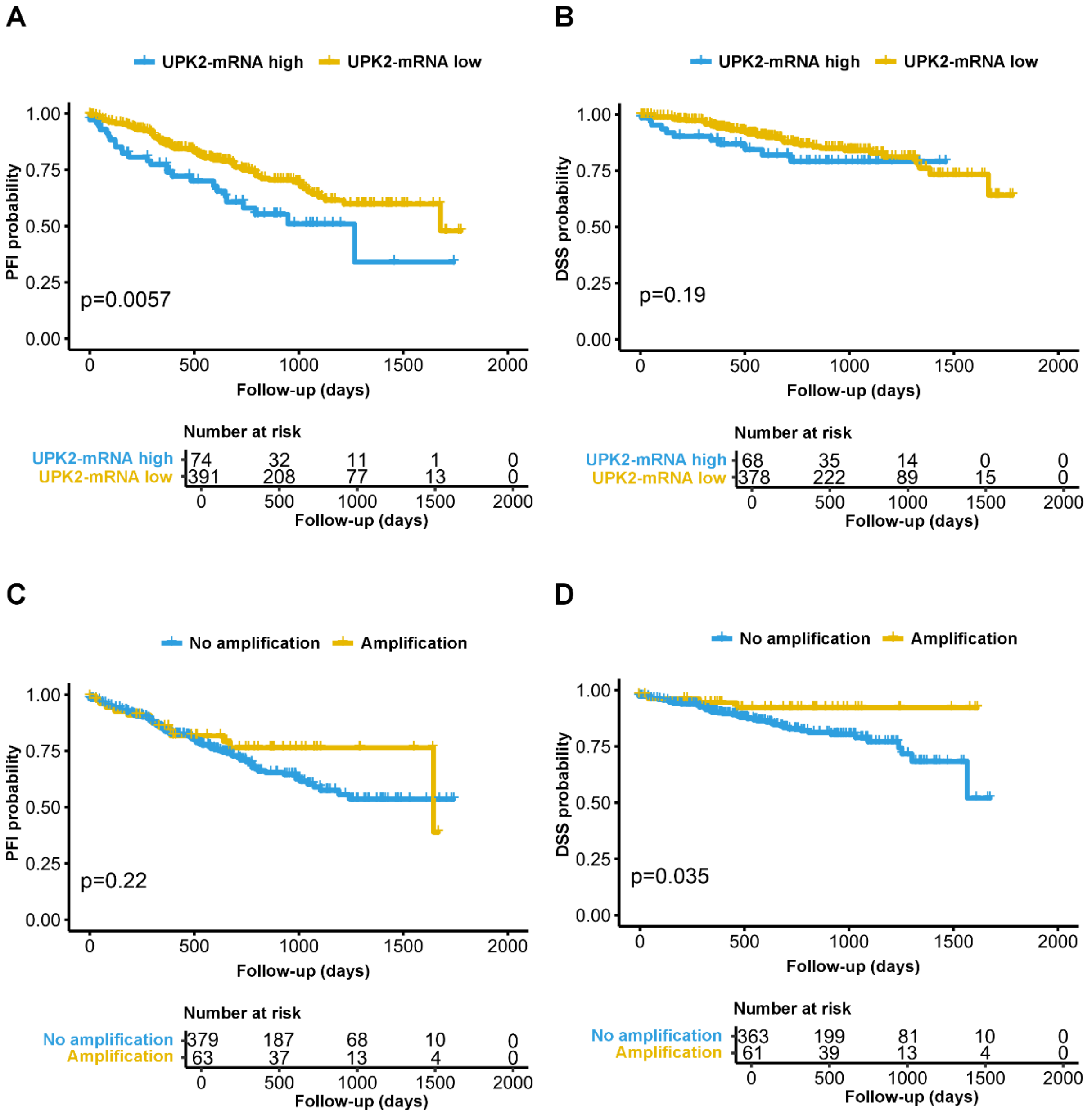

Figure S9. Kaplan-Meier survival analyses in The Cancer Genome Atlas cohort according to *UPK2* mRNA levels and locus amplification. (A,B) The association of UPK2 mRNA levels with progression-free interval (A) and disease-specific survival (B). (C,D) The association of *UPK2* locus (11q23.3) amplification with progression-free interval (C) and disease-specific survival (D).

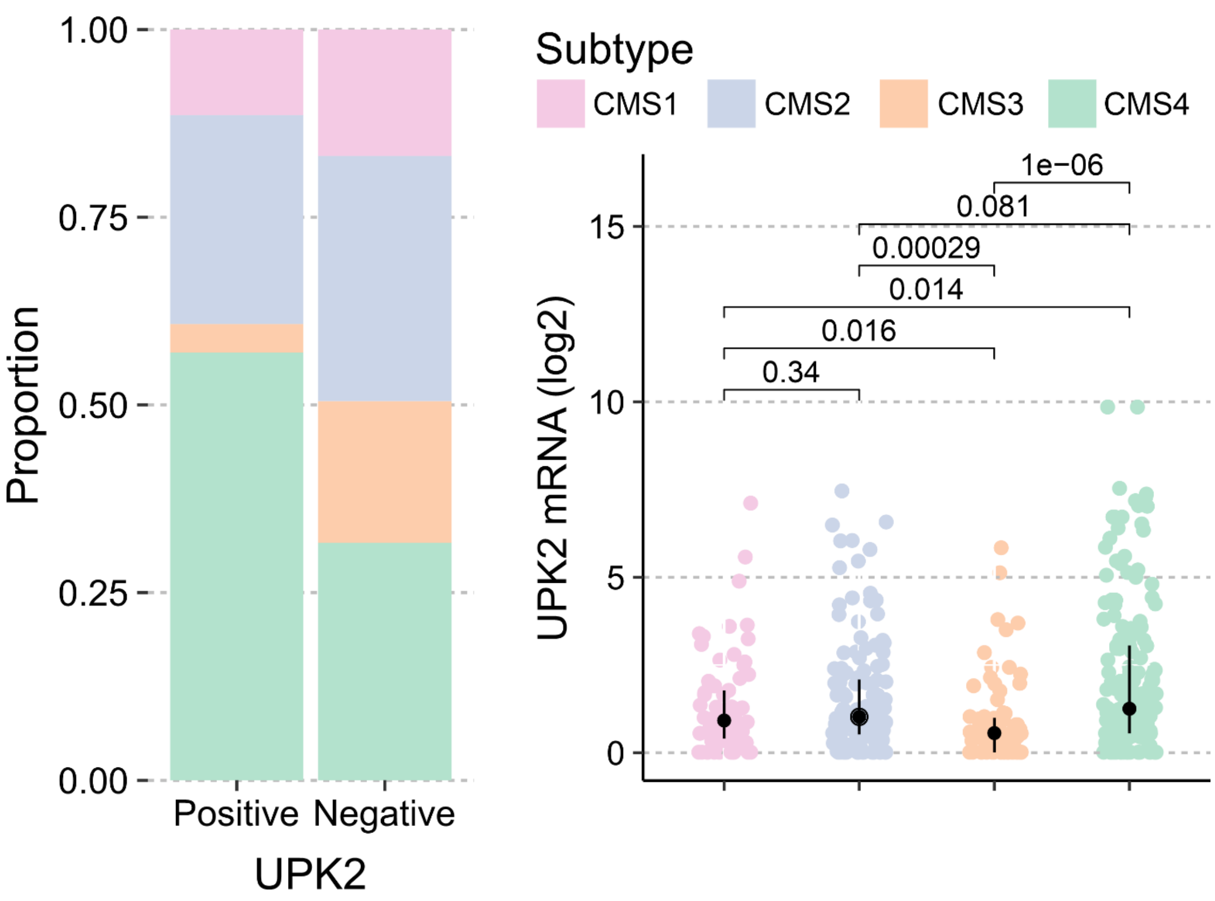

Figure S10. Consensus Molecular Subtype classification of UPK2-positive versus UPK2-negative colorectal cancers in The Cancer Genome Atlas cohort.

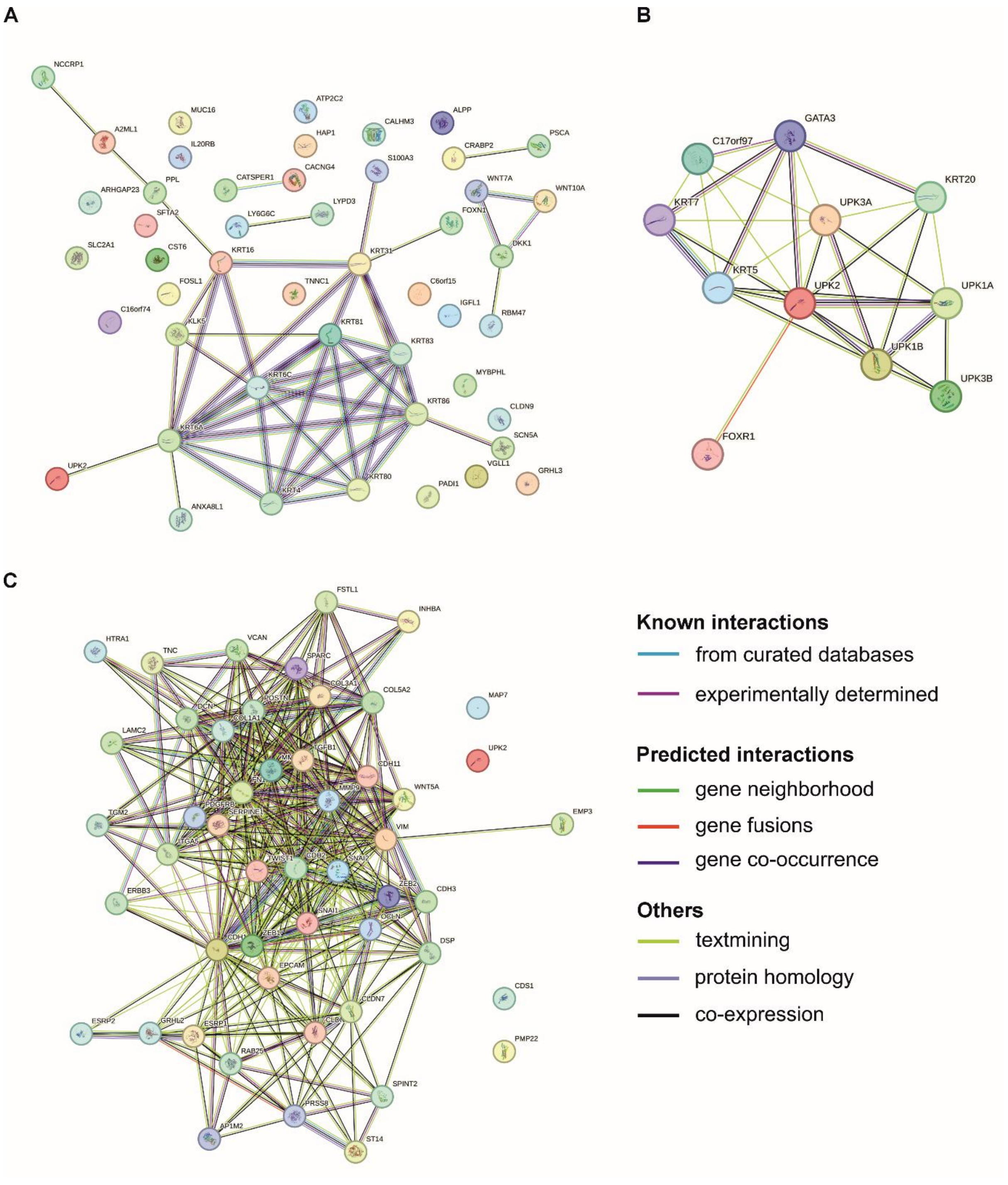

Figure S11. STRINGdb protein-protein association analysis of UPK2. (A) The associations of UPK2 with the 50 most differentially expressed genes in UPK2-positive (vs. UPK2-negative) colorectal cancers. A significant interaction was seen between UPK2 (red dot) and KRT6A. (B) The associations of UPK2 displayed after a single gene input (*UPK2*). Associations with additional keratins were seen. (C) The associations of UPK2 with 50 epithelial-mesenchymal transition involved genes selected from the EMTome. No significant interactions were identified for UPK2.
